# Intestinal Gastrin/CCKBR Axis Protects against Type 2 Diabetes by Reducing Intestinal Glucose Absorption through the PI3K/Akt/eIF4B Signaling Pathway

**DOI:** 10.1002/advs.202410032

**Published:** 2025-02-14

**Authors:** Xue Liu, Xing Liu, Yunpeng Liu, Anxiong Long, Wei Liu, Shiyun Sun, Shuaibing Lu, Xianxian Wu, Xiaodi Jia, Pedro A Jose, Qiang Wei, Xiaoliang Jiang, Haizeng Zhang, Zhiwei Yang

**Affiliations:** ^1^ Institute of Laboratory Animal Sciences (CAMS & PUMC) National Center of Technology Innovation for Animal Model National Human Diseases Animal Model Resource Center NHC Key Laboratory of Human Disease Comparative Medicine Beijing Engineering Research Center for Experimental Animal Models of Human Critical Diseases 100021 Beijing China; ^2^ Department of Cardiology the Second Affiliated Hospital School of Medicine Zhejiang University State Key Laboratory of Transvascular Implantation Devices Heart Regeneration and Repair Key Laboratory Zhejiang Province 310009 Hangzhou China; ^3^ Graduate School of Hebei North University Zhangjiakou Hebei 075031 China; ^4^ Department of Colorectal Surgery National Cancer Center/National Clinical Research Center for Cancer/Cancer Hospital Chinese Academy of Medical Sciences and Peking Union Medical College 100021 Beijing China; ^5^ State Key Laboratory of Molecular Oncology National Cancer Center/National Clinical Research Center for Cancer/Cancer Hospital Chinese Academy of Medical Sciences and Peking Union Medical College Beijing 100021 China; ^6^ Taihe County People's Hospital The Taihe Hospital of Wannan Medical College 21 Jiankang Road Taihe County Anhui Province 236600 China; ^7^ Department of Pharmacology and Physiology The George Washington University School of Medicine & Health Sciences Washington DC 20052 USA; ^8^ Department of Medicine Division of Kidney Diseases & Hypertension The George Washington University School of Medicine & Health Sciences Washington DC 20052 USA

**Keywords:** cholecystokinin B receptor, Gastrin‐SiO_2_ microspheres, glucose transporter type 2, sodium/glucose cotransporter 1, type 2 diabetes

## Abstract

The Gastrin/CCKBR axis is essential for inhibiting intestinal sodium absorption, but its effects on intestinal glucose metabolism remain elusive. This study aims to determine the role of intestinal Gastrin/CCKBR on glucose absorption in the development of type 2 diabetes (T2D). Intestinal epithelial cell‐specific *Cckbr* knockout mice and control wild‐type mice are fed normal diet (ND, 10% fat) or high fat diet (HFD, 60% fat) to study the effect of intestinal Gastrin/CCKBR on blood glucose levels. Gastrin‐SiO_2_ microspheres (20 mg kg^−1^ d^−1^) are designed so that gastrin specifically stimulates intestinal CCKBR, without its absorption into the circulation. Mice with silenced intestinal *Cckbr* has pre‐diabetes mellitus (Pre‐DM) that rapidly progressed into T2D when fed HFD. Moreover, Gastrin‐SiO_2_ microspheres markedly reduce glucose absorption in duodenum obtained from patients with T2D. In mice with HFD‐induced T2D, Gastrin‐SiO_2_ microspheres reduce intestinal glucose absorption by down‐regulating intestinal SGLT1 and GLUT2 expressions and stimulating incretin secretion. This study shows the important role of intestinal Gastrin/CCKBR in intestinal glucose absorption. Gastrin‐SiO_2_ microspheres may be a promising strategy for the treatment of patients with T2D.

## Graphical abstract

In intestinal epithelial cells, Gastrin/CCKBR stimulation prevents high glucose‐stimulated SGLT1 (SLC5A1) and GLUT2 (SLCA2) expressions, via the PI3K/AKT/eIF4B signaling pathway, with facilitatory effects on the secretion of incretins, which could be therapeutic targets for clinical treatment in T2D.

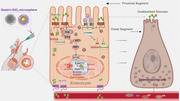

## 1 Introduction

Type 2 diabetes (T2D) is the most prevalent progressive metabolic disorder, affecting more than 425 million people worldwide.^[^ ^1^ ^]^ The risk factors for T2D consist of genetic predisposition, lifestyle, and unhealthy diet, among which high glucose intake is a common factor.^[^ ^2^ ^]^ Excessive dietary sugar consumption increases postprandial glucose level (≥8.6 mmol L^−1^, 1‐h post‐glucose load), which is an independent risk factor for T2D and its life‐threatening complications.^[^ ^3^, ^4^, ^5^ ^]^ The World Health Organization (WHO) recommends that the daily sugar intake should be less than 10% of total energy (≈25 g for an adult). However, worldwide glucose intakes range from 13.5% to 24.6% in adults and up to 19% of total energy in children and adolescents, which are much higher than the WHO recommendation.^[^ ^6^ ^]^ Therefore, interventions to inhibit the intestinal absorption of glucose to mitigate the deleterious consequences of “inappropriate” sugar intake may be an effective therapy for T2D.

CCKBR (cholecystokinin B receptor) is highly expressed in the brush border membrane of the intestine (Genotype‐Tissue Expression (GTEx) and Functional Annotation of the Mammalian Genome 5 (FANTOM.5)) and the kidney, activation of which decreases intestinal sodium absorption and renal tubular sodium reabsorption.^[^ ^7^, ^8^ ^]^ In the intestines, the absorption of glucose and water is predominately driven by sodium uptake.^[^ ^9^ ^]^ Concomitant with the decrease in intestinal sodium absorption, the sodium/glucose cotransporter 1 (SGLT1)‐mediated glucose uptake is also reduced in the small intestine.^[^ ^10^, ^11^ ^]^ In mammals, SGLT1‐mediated dietary glucose absorption in the gastrointestinal tract into the bloodstream mainly occurs in the small intestines, which has become an important target for the prevention and treatment of diabetes.^[^ ^12^, ^13^ ^]^ However, the marked decrease in intestinal absorption of glucose and galactose resulting from the complete inhibition of intestinal SGLT1 causes severe diarrhea which greatly limits its clinical application in humans.^[^ ^12^, ^13^, ^14^, ^15^ ^]^ Indirect and moderate inhibition of SGLT1‐induced intestinal glucose transport, via Gastrin/CCKBR, could be a novel treatment for T2D.

Intestinal CCKBR can be stimulated by gastrin, which is secreted by antral G cells in the stomach.^[^ ^16^, ^17^ ^]^ In animal models of diabetes, improved glucose control and neogenesis of functional β‐cell mass occur after gastrin treatment.^[^ ^18^, ^19^, ^20^, ^21^, ^22^, ^23^ ^]^ Gastrin, via CCKBR, significantly reduced glucose absorption in human intestinal epithelial cells (HIECs) (based on our preliminary exploratory study). Therefore, we hypothesized that intestinal CCKBR may be a key driver to inhibit, partially, intestinal glucose absorption; indirect SGLT1 inhibition could be a novel strategy for the treatment of T2D.

In this study, we showed that the reduction of the expression of CCKBR in small intestines is concomitant with impaired glucose metabolism in both patients and mice with T2D. In intestinal epithelial cell‐specific *Cckbr*‐knockout mice, intestinal glucose metabolism dysfunction occurs by the upregulation of intestinal SGLT1 and GLUT2 expression and decrease in the secretion of incretin hormones. Orally administered Gastrin‐SiO_2_ microspheres (Chinese patent number: ZL 2021 1 0223615.0), by stimulating intestinal CCKBR, may provide an effective strategy in the treatment of T2D in a mouse model by inhibiting the PI3K/Akt/eIF4B‐mediated SGLT1/GLUT2 pathway. To ascertain the applicability of our findings in T2D mice to humans, we studied the effect of Gastrin‐SiO_2_ microspheres in primary cultures IECs obtained from healthy donors and patients with T2D. We found that glucose absorption was increased to a greater extent in IECs from T2D patients than healthy donors, which was decreased by Gastrin‐SiO_2_ microspheres. Taken together, these data identified Gastrin/CCKBR axis as a critical regulator of intestinal glucose metabolism, and suggest that Gastrin‐SiO_2_ microspheres, administered orally, may be a viable therapeutic approach in the management of T2D.

## 2 Results

### 2.1 Intestinal CCKBR Levels Are Reduced in Patients and Mice with T2D

**Figure 1:**
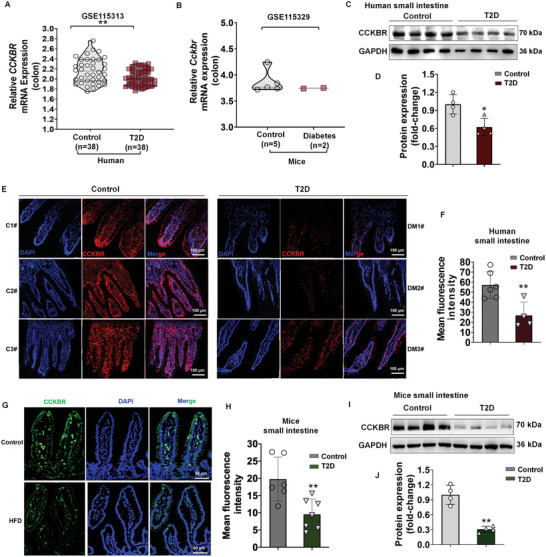
Intestinal CCKBR expression is decreased in patients and mice with T2D. A) *CCKBR* mRNA expression in patients with colon cancer, with and without T2D (*n* = 38 per group). The mRNA microarray data were obtained from the GEO database (Accession No: GSE115313), B) *Cckbr* mRNA expression in the colon tumor xenografts in streptozotocin‐induced diabetic mice (Control: *n* = 5, Diabetes: *n* = 2). The mRNA microarray data were obtained from the GEO database (Accession No: GSE115329). C,D) Western blots and quantification of CCKBR in the small intestine (duodenum) of normal humans and T2D patients (*n* = 4 per group). E) Immunofluorescence in the small intestine (duodenum) of normal humans and T2D patients. (CCKBR, red; DAPI, blue); F) Quantification of the mean fluorescence intensity using ImageJ software. (Control: *n* = 6; T2D: *n* = 4); G) Immunofluorescence in the small intestine (duodenum) of control mice (fed normal diet, 10% fat, *n* = 6) and HFD‐induced T2D mice (fed high fat diet, 60% fat, *n* = 7). (CCKBR, green; DAPI, blue); H) Quantification of the mean fluorescence intensity using ImageJ software. I,J) Western blots and quantification of CCKBR (*n* = 4 per group). All data are expressed as mean ± SEM. Unpaired Student's *t*‐test, Control versus T2D, Control versus Diabetes *p < 0.05, **p < 0.01.

To determine the clinical relevance of CCKBR in T2D, we initially analyzed the microarray datasets available from the Gene Expression Omnibus database. In a cohort of patients with colon cancer, with and without T2D (Accession No: GSE115313), the diabetic intestine had decreased *CCKBR* mRNA (**Figure** **1**A). In a colon tumor xenograft model in streptozotocin‐induced diabetic mice (Accession No: GSE115329), *Cckbr* expression in the colon was lower in mice with T2D than mice without T2D (Figure 1B). We then focused on the CCKBR expression in the intestines from human clinic participants with T2D and healthy human donors. The basic information on the participants is listed in **Table** **1**. Quantitative protein expression analysis showed a significant decrease in CCKBR expression in patients with T2D compared with the healthy controls (Figure 1C,D, and Figure S1A, Supporting Information and Figure 1B). As shown in Figure 1E,F and Figure S1C (Supporting Information) and Figure 1D, strong CCKBR (red) expression was observed in normal human small and large intestines, but CCKBR expression was significantly reduced in the small and large intestines from T2D patients. We also examined the small and large intestinal CCKBR expression in established experimental models of diabetes, e.g., T2D induced by high‐fat diet (HFD). Immunofluorescence and western blots showed significantly decreased CCKBR expression in the T2D mice compared with control mice (Figure 1G–J and Figure S1E–H, Supporting Information). These results showed that the expression of intestinal CCKBR is reduced in patients and mice with T2D.

**Table 1:** Information on non‐diabetic and diabetic patients. F, female; M, male. GLU: glucose, GA: Glycated hemoglobin, TG: Triglycerides, HDL‐CHO: High‐density lipoprotein cholesterol, LDL‐CHO: Low‐density lipoprotein cholesterol, ALT: Alanine aminotransferase, AST: Aspartate aminotransferase, LDH: Lactate dehydrogenase, CK: Creatine kinase, CK‐MB: Creatine kinase‐MB isoenzyme.

Variables	Non‐diabetic [*n* = 6]	Diabetic [*n* = 4]	*p* value
Age [years]	63.0 ± 10.1	60.5 ± 6.6	0.63
Sex	4M, 2F	3M, 1F	–
GLU [mmol L^−1^]	5.4 ± 0.5	8.3 ± 3.1	0.04*
BMI	23.9 ± 2.0	24.2 ± 3.2	0.92
GA [%]	14.9 ± 0.6	19.5 ± 7.3	0.05*
TG [mmol L^−1^]	1.6 ± 0.7	1.2 ± 0.4	0.41
HDL‐CHO [mmol L^−1^]	1.1 ± 0.3	1.1 ± 0.1	0.91
LDL‐CHO [mmol L^−1^]	3.0 ± 0.8	3.1 ± 1.2	0.82
ALT [U L^−1^]	15.5 ± 5.5	26.4 ± 15.0	0.13
AST [U L^−1^]	18.8 ± 3.6	23.7 ± 6.2	0.15
LDH [U L^−1^]	163.6 ± 28.8	155.7 ± 16.2	0.63
CK [U L^−1^]	69.5 ± 30.4	50.8 ± 14.9	0.29
CK‐MB [U L^−1^]	10.2 ± 3.6	12.9 ± 1.2	0.19

### 2.2 Intestinal Epithelial Cell *Cckbr*‐Silenced Mice Display the Pre‐Diabetes Mellitus (Pre‐DM) Phenotype

**Figure 2:**
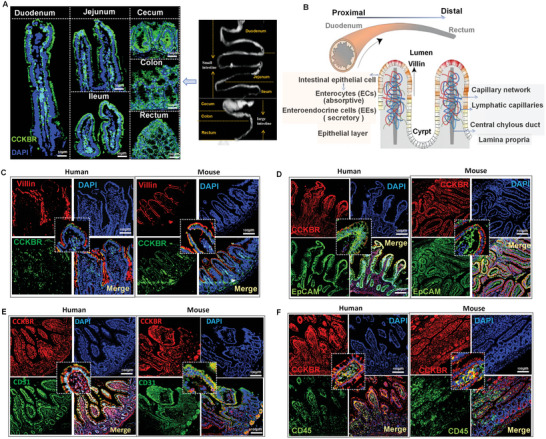
CCKBR expression in the intestines of human and mouse. A) Immunofluorescence in the small intestine (duodenum, jejunum, ileum) and large intestine (cecum, colon, rectum) in *Villin‐Cckbr^+/+^* mice (CCKBR, green; DAPI, blue); B) Illustration of the proximal‐to‐distal axis of the intestine and villi; C) Immunofluorescence of villin (red) and CCKBR (green), D) EpCAM (green) and CCKBR (red), E) CD31 (green) and CCKBR (red), F) CD45 (green) and CCKBR (red) in the small intestine (duodenum) from humans and *Villin‐Cckbr^+/+^* mice.

Our previous study showed that CCKBR is expressed in human and mouse intestines.^[^ ^7^ ^]^ We quantified CCKBR expression in the different segments of mouse small and large intestines, namely the duodenum, jejunum, ileum, cecum, colon, and rectum. Immunofluorescence assay showed that CCKBR is abundantly expressed in the villi and basolateral membranes of the small and large intestinal segments (**Figure** **2**A). As shown in Figure 2B, the epithelial layer of the villi is composed of absorptive and secretory epithelial cells (e.g., L cells). The lamina propria at the center of the villi is dense, containing blood vessels of the villous capillary network, lymphatic capillaries, and central chylous duct. We examined the expression of CCKBR in different intestinal cells. Immunofluorescence was further used to confirm the localization of villin (red) and CCKBR (green) (Figure 2C), EpCAM (green), and CCKBR (red) (Figure 2D), CD31 (green) and CCKBR (red) (Figure 2E), and CD45 (green) and CCKBR (red) (Figure 2F), in the intestines of humans and mice. CCKBR was expressed in the intestinal epithelial cells, villi (identified by EpCAM), endothelial cells (identified by CD31), and lymphocytes (identified by CD45). Glucose absorption mainly occurs at the intestinal epithelial cells, following enzymatic digestion of carbohydrates. To verify our conjecture about the regulatory effects of CCKBR on glucose absorption, we generated intestinal epithelial cell‐specific conditional *Cckbr‐*knockout (*Villin‐Cckbr^−/−^*) mice; wild‐type (*Villin‐Cckbr^+/+^*) mice were used as controls.

**Figure 3:**
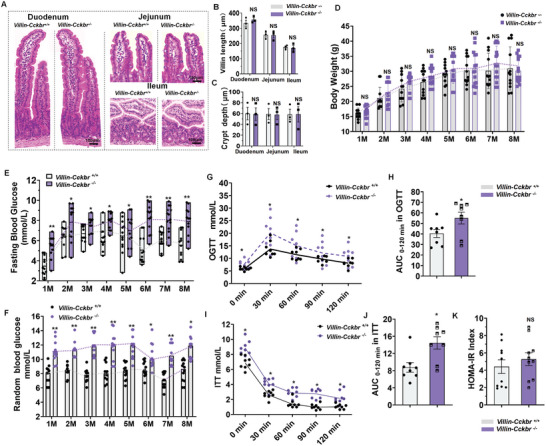
Intestinal epithelial cells in *Villin‐Cckbr^−/−^* mice display the prediabetes (Pre‐DM) phenotype. A) HE staining of the duodenum, jejunum, and ileum of *Villin‐Cckbr^+/+^* (*n* = 3) and *Villin‐Cckbr^−/−^* mice (*n* = 3). B) Villi length (µm); C) Crypt depth (µm); and D) Body weights of *Villin‐Cckbr^+/+^* and *Villin‐Cckbr^−/−^* mice from 1–8 mo (M) of age, *Villin‐Cckbr^+/+^* (*n* = 12) and *Villin‐Cckbr^−/−^* mice (*n* = 15). All data are expressed as mean ± SEM, two‐way ANOVA, post hoc Scheffe test (D), *Villin‐Cckbr^+/+^* mice versus *Villin‐Cckbr^−/−^* mice, NS: Not significant; 8 m‐*Villin‐Cckbr^+/+^* mice versus 1 m‐*Villin‐Cckbr^+/+^* mice, 8 m‐*Villin‐Cckbr^−/−^* mice versus 1 m‐*Villin‐Cckbr^−/−^* mice, ***p < 0.0001, 8 m‐*Villin‐Cckbr^+/+^* mice versus 2 m‐*Villin‐Cckbr^+/+^* mice, 8 m‐*Villin‐Cckbr^−/−^* mice versus 2 m‐*Villin‐Cckbr^−/−^* mice, *p < 0.05, 8 m‐*Villin‐Cckbr^+/+^* mice versus 3 m‐*Villin‐Cckbr^+/+^* mice, 8 m‐*Villin‐Cckbr^−/−^* mice versus 3 m‐*Villin‐Cckbr^−/−^* mice,*p < 0.05; E) Fasting blood glucose levels of *Villin‐Cckbr^+/+^* and *Villin‐Cckbr^−/−^* mice from 1–8 m of age, *Villin‐Cckbr^+/+^* and *Villin‐Cckbr^−/−^* mice, (*n* = 12/group), two‐way ANOVA, post hoc Scheffe test, *Villin‐Cckbr^+/+^* mice versus *Villin‐Cckbr^−/−^* mice, *p < 0.05, **p < 0.01; 8 m‐*Villin‐Cckbr^+/+^* mice versus 1 m‐*Villin‐Cckbr^+/+^* mice, 8 m‐*Villin‐Cckbr^−/−^* mice versus 1 m‐*Villin‐Cckbr^−/−^* mice,*p < 0.05; F) Random blood glucose levels of *Villin‐Cckbr^+/+^* and *Villin‐Cckbr^−/−^* mice from 1–8 m of age, *Villin‐Cckbr^+/+^* (*n* = 12) and *Villin‐Cckbr^−/−^* mice (*n* = 12), two‐way ANOVA, post hoc Scheffe test, *Villin‐Cckbr^+/+^* mice versus *Villin‐Cckbr^−/−^* mice, *p < 0.05, **p < 0.01; 8 m‐*Villin‐Cckbr^+/+^* mice versus 1M‐*Villin‐Cckbr^+/+^* mice, 8 m‐*Villin‐Cckbr^−/−^* mice versus 1 m‐*Villin‐Cckbr^−/−^* mice, NS: Not significant; G) Oral glucose tolerance test (OGTT) in 8 mo‐old *Villin‐Cckbr^+/+^* (*n* = 8) and *Villin‐Cckbr^−/−^* mice (*n* = 8); H) OGTT area under the curve (AUC) in 8 mo‐old *Villin‐Cckbr^+/+^* and *Villin‐Cckbr^−/−^* mice; I) Insulin tolerance test (ITT) in 8 mo‐old *Villin‐Cckbr^+/+^* (*n* = 8) and *Villin‐Cckbr^−/−^* mice (*n* = 8); J) ITT area under the curve (AUC) in 8 mo‐old *Villin‐Cckbr^+/+^* and *Villin‐Cckbr^−/−^* mice; K) HOMA‐IR index (fasting glucose levels × fasting insulin levels)/22.5 in 8 mo‐old *Villin‐Cckbr^+/+^* (*n* = 10) and *Villin‐Cckbr^−/−^* mice (*n* = 10). All data are expressed as mean ± SEM, two‐way ANOVA, post hoc Scheffe test (D–G, I), Unpaired Student's *t*‐test (B, C, H–K), *Villin‐Cckbr^+/+^* mice versus *Villin‐Cckbr^−/−^* mice, *p < 0.05, **p < 0.01, NS: Not significant.

Intestinal epithelial cell‐specific deletion of *Cckbr*, verified using agarose gel electrophoresis, RT‐PCR, and western blot (Figure S2A–D, Supporting Information), did not influence the intestine morphology (including villi lengths and crypts length) (**Figure** **3**A–C) and body weight (Figure 3D). The fasting blood glucose levels were higher in *Villin‐Cckbr^−/−^* mice than in *Villin*‐*Cckbr^+/+^* mice (Figure 3E), and a similar trend was observed for the random blood glucose levels (Figure 3F). *Villin‐Cckbr^−/−^* mice also had higher blood glucose levels than *Villin‐Cckbr^+/+^* mice during the oral glucose tolerance test (OGTT) and insulin tolerance test (ITT) (*p* < 0.05); the area under the curve (AUC) and random blood glucose levels were also higher in *Villin‐Cckbr^−/−^* than *Villin‐Cckbr^+/+^* mice (Figure 3G–J) (*p* < 0.05). HOMA‐insulin resistance was not different between the two groups (Figure 3K). These data suggested that mice with intestinal epithelial cell‐specific conditional *Cckbr* knockout had pre‐diabetes mellitus.

### 2.3 Intestinal Epithelial Cell *Cckbr*‐Silenced Mice Fed HFD Diet Rapidly Develop T2D

**Figure 4:**
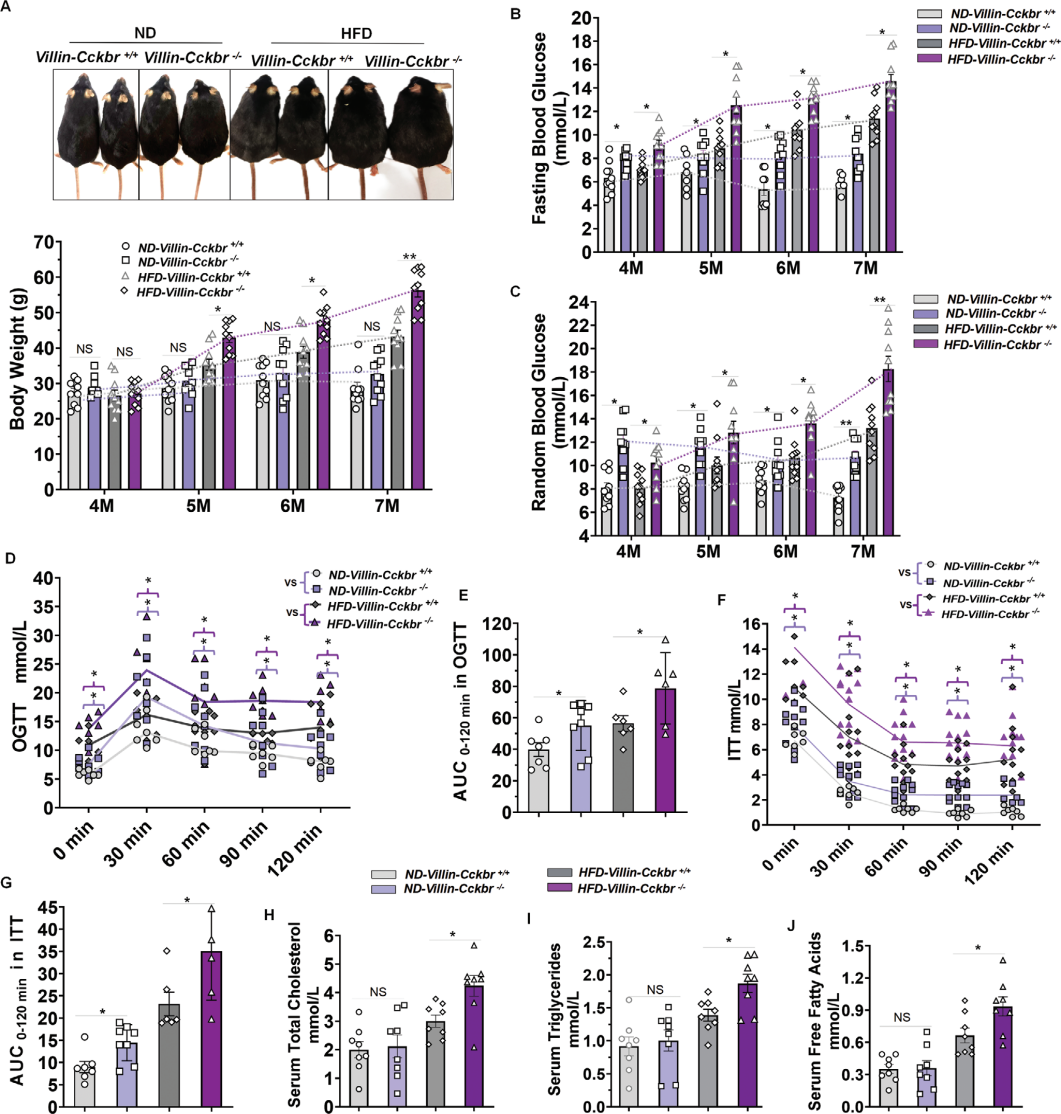
*Villin Cckbr* ^−^ *^/^* ^−^ fed HFD diet rapidly develops T2D. A) Photographs and body weights of normal fat diet (ND, 10% fat)‐fed mice: *ND‐Villin‐Cckbr^+/+^* (*n* = 10) and *ND‐Villin‐Cckbr* ^−^ *^/^* ^−^ mice (*n* = 10), and high fat diet (HFD, 60% fat)‐fed mice: *HFD‐Villin‐Cckbr^+/+^* (*n* = 10) and *HFD‐Villin‐Cckbr* ^−^ *^/^* ^−^ mice (*n* = 10); B,C) Fasting and random blood glucose levels in ND‐fed and HFD‐fed 4–7 mo‐old mice (*n* = 10 per group). D) Oral glucose tolerance test (OGTT) in ND‐fed and HFD‐fed 7 mo‐old mice (*n* = 6–8 per group). E) OGTT area under the curve (AUC) from (D). F) Insulin tolerance test (ITT) in ND‐fed and HFD‐fed 7‐mo‐old mice (*n* = 6 per group). G) Area under the curve (AUC) from ITT plot from (F). H) Serum total cholesterol, I) serum triglycerides, and J) serum free fatty acids in ND‐fed and HFD‐fed 7 mo‐old mice (*n* = 8 per group). All data are expressed as mean ± SEM, three‐way ANOVA, post hoc Scheffe test (A–D and F), *ND‐Villin‐Cckbr^+/+^* mice versus *ND‐Villin‐Cckbr* ^−^ *^/^* ^−^ mice, *HFD‐Villin‐Cckbr^+/+^* mice versus *HFD‐Villin‐Cckbr* ^−^ *^/^* ^−^ mice, *p < 0.05, **p < 0.01, NS: Not significant. 7 m‐*ND‐Villin‐Cckbr^+/+^* mice versus 4 m‐*ND‐Villin‐Cckbr^+/+^* mice, NS: Not significant; 7 m‐*ND‐Villin‐Cckbr* ^−^ *^/^* ^−^ mice versus 4 m‐*ND‐Villin‐Cckbr* ^−^ *^/^* ^−^ mice, NS: Not significant; 7 m‐*HFD‐Villin‐Cckbr^+/+^* mice versus 4 m‐*HFD‐Villin‐Cckbr^+/+^* mice, *p < 0.05; 7 m‐*HFD‐Villin‐Cckbr* ^−^ *^/^* ^−^ mice versus 4 m‐*HFD‐Villin‐Cckbr* ^−^ *^/^* ^−^ mice, ***p < 0.01; two‐way ANOVA, post hoc Scheffe test (E and G–J), *ND‐Villin‐Cckbr^+/+^* mice versus *ND‐Villin‐Cckbr* ^−^ *^/^* ^−^ mice, *HFD‐Villin‐Cckbr^+/+^* mice versus *HFD‐Villin‐Cckbr* ^−^ *^/^* ^−^ mice, *p < 0.05, **p < 0.01, NS: Not significant.

To ascertain the role of intestinal epithelial cell *Cckbr* in T2D, 4‐mo old *Villin‐Cckbr^+/+^* and *Villin‐Cckbr^−/−^* mice were fed either normal‐fat diet (ND, 10% fat) or high‐fat diet (HFD, 60% fat) for 3 months. ND‐fed *Villin‐Cckbr^−/−^* and ND‐fed *Villin‐Cckbr^+/+^* mice had comparable body weights and levels of serum total cholesterol, triglycerides, and free fatty acids (**Figure** **4**A,H–J). However, the fasting and random blood glucose levels were higher in ND‐fed *Villin‐Cckbr^−/−^* mice than ND‐fed *Villin‐Cckbr^+/+^* mice (Figure 4B,C). ND‐fed *Villin‐Cckbr^−/−^* mice also had higher blood glucose levels than ND‐fed *Villin‐Cckbr^+/+^* mice during the OGTT and ITT (Figure 4D–G). In contrast to ND‐fed *Villin‐Cckbr^−/−^* mice, HFD‐fed *Villin‐Cckbr^−/−^* mice had a faster and greater increase in body weight and higher fasting and random blood glucose levels (Figure 4A–C) than HFD‐fed *Villin‐Cckbr^+/+^* mice, with abnormal OGTT and ITT (Figure 4D–G). The serum total cholesterol, triglycerides, and free fatty acid levels were also higher in HFD‐*Villin‐Cckbr* ^−^ *^/^* ^−^ mice than HFD‐*Villin‐Cckbr^+/+^* mice (Figure 4H–J). These results show that intestine epithelial cell‐specific deletion of *Cckbr* induced the rapid development of T2D in these HFD‐fed mice.

### 2.4 Gastrin‐SiO_2_ Microspheres Mitigate the Glucose Metabolic Dysfunction in Mice with T2D

**Figure 5:**
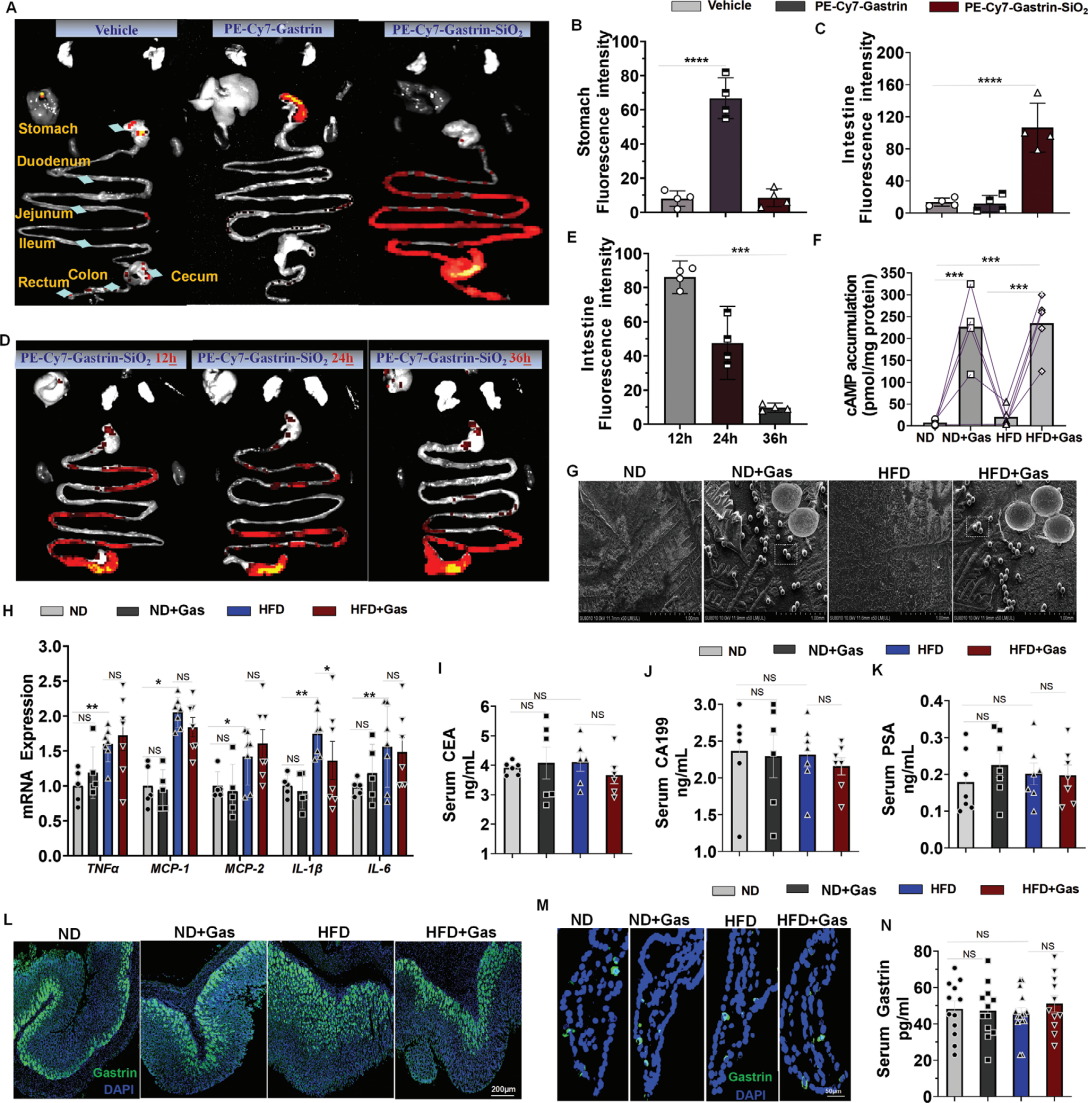
Gastrin‐SiO_2_ microspheres intestinally targeted to stimulate CCKBR effectively and biocompatible do not cause obvious adverse side effects. A) In vivo fluorescence imaging and fluorescence of gastrin in the B) stomach and C) intestines, quantified by ImageJ in the three groups of mice; D) Fluorescence intensity measured by fluorescence emission tomography at 12, 24, and 36 h after C57BL/6J mice were gavaged with PE‐cy7‐Gastrin‐SiO_2_; E) Fluorescence intensity calculated by ImageJ in the intestine (duodenum). All data are expressed as mean ± SEM, one‐way Student's *t*‐test (B, C, E), PE Cy7‐Gastrin versus Vehicle; PE Cy7‐Gastrin‐SiO_2_ versus Vehicle, ****p < 0.0001; F) cAMP accumulation in intestinal epithelial cells from C57BL/6J mice fed normal diet (ND, 10% fat) gavaged with saline, ND‐fed C57BL/6J mice gavaged with Gastrin‐SiO_2_ microspheres (ND+Gas), HFD‐fed C57BL/6J (HFD, 60% fat) gavaged with saline, and HFD‐fed C57BL/6J mice gavaged with Gastrin‐SiO_2_ microspheres (HFD+Gas), *n* = 4 per group; G) Fecal samples from ND mice (*n* = 3), ND‐fed mice gavaged with gastrin‐SiO_2_ microspheres (*n* = 3), HFD‐fed mice (*n* = 3), and HFD‐fed mice gavaged with gastrin‐SiO_2_ microspheres (*n* = 3) were visualized by SEM (scanning electron microscopy); H) mRNA expressions of TNFα (tumor necrosis factor α), MCP‐1 (monocyte chemotactic protein‐1), MCP‐2 (monocyte chemotactic protein‐2), IL‐1β (interleukin‐1β), and IL‐6 (interleukin‐6) in small intestine (duodenum) from all groups (ND: *n* = 6, ND+Gas: *n* = 6, HFD: *n* = 7, and HFD+Gas: *n* = 7); I) Serum concentrations of colon cancer markers CEA (carcinoembryonic antigen), J) CA199 (carbohydrate antigen199), and K) PSA (prostate specific antigen) in all groups (*n* = 5–7 per group); L) Immunofluorescence of gastrin (green) in stomach and M) small intestine (duodenum), *n* = 3 per group; N) Serum gastrin levels mice from all groups (*n* = 11–15 per group). All data are expressed as mean ± SEM, two‐way ANOVA, post hoc Scheffe test (F and H–K, N), HFD mice versus ND mice, HFD+Gas mice versus HFD mice, ND+Gas mice versus ND mice, *p < 0.05, **p < 0.01, ***p < 0.001, NS: not significant.

To further assess the potential role of intestinal epithelial cell CCKBR in the progression of T2D, Gastrin‐SiO_2_ microspheres (Chinese patent number: ZL 2021 1 0223615.0) were designed to activate CCKBR in the intestinal epithelial cells of HFD‐induced diabetic mice. As shown in **Figure** **5**A,B, gastrin (without SiO_2_ microspheres, PE‐Cy7‐gastrin, a gastrin polyclonal antibody conjugated with Cy7 dye) bound to CCKBR in the stomach but not to CCKBR in the intestines. When gastrin was conjugated with microspheres (PE‐Cy7‐Gastrin SiO_2_), gastrin efficiently bound to the intestinal CCKBR in the C57BL/6J mice (12–36 h) (Figure 5A,C,D,E) and showed strong, selective, small intestinal CCKBR activation, proved by the increase in cAMP accumulation (Figure 5F). We have reported that Gastrin‐SiO_2_ microspheres, which have an average particle size of 70 µm, can efficiently stimulate intestinal CCKBR, but cannot be absorbed from the digestive tract into the bloodstream, thereby avoiding systemic side effects.^[^ ^7^ ^]^ The scanning electron microscopy (SEM) images show SiO_2_ microspheres in fecal samples from oral Gastrin‐SiO_2_ microspheres‐treated mice (Figure 5G). Gastrin‐SiO_2_ microspheres (20 mg kg^−1^ day^−1^, 80 µL/10 g), administered by gavage (10–20 s), did not affect the mRNA expression of inflammatory factors (TNFα, MCP‐1, MCP‐2, IL‐1β, and IL‐6) in the intestine (Figure 5H) and colon cancer promoting markers in the serum (CEA, CA199, and PSA) (Figures 5I–K). Gastrin is predominantly secreted by G‐cells in the stomach and duodenum^[^ ^24^ ^]^; its expression is shown in the stomach (Figure 5L; Figure S3A, Supporting Information), duodenum (Figure 5M; Figure S3B, Supporting Information), and serum (Figure 5N). Endogenous gastrin secretion was not affected by Gastrin‐SiO_2_ microsphere treatment (Figure 5L–N). These results showed that Gastrin‐SiO_2_ microspheres are intestinally targeted, bio‐compatible without obvious adverse side effects, and efficiently stimulate CCKBR.

**Figure 6:**
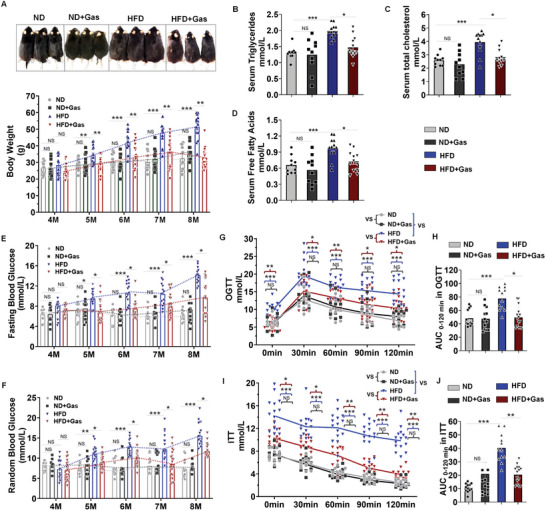
Gastrin‐SiO_2_ microspheres mitigate glucose metabolic dysfunction in T2D mice. A) Representative photographs and body weights of C57BL/6J mice fed normal diet (ND, 10% fat, *n* = 12), ND diet + Gastrin‐SiO_2_ microspheres (ND+Gas, 20 mg kg^−1^ d^−1^, *n* = 12), high fat diet (HFD, 60% fat, *n* = 15), or HFD diet + Gastrin‐SiO_2_ microspheres (HFD+Gas, 20 mg kg^−1^ d^−1^, *n* = 20). B) Serum levels of triglycerides, C) total cholesterol, and D) free fatty acids were measured by ELISA (*n* = 10–12 per group). E) Fasting and F) random blood glucose levels of 4–8‐mo‐old mice (ND: *n* = 12; ND+Gas: *n* = 12; HFD: *n* = 15; HFD+Gas: *n* = 20). G) Oral glucose tolerance test (OGTT) (ND: *n* = 12; ND+Gas: *n* = 12; HFD: *n* = 15; HFD+Gas: *n* = 20). H) Area under the curve (AUC) of OGTT plots from (G). I) Insulin tolerance test (ITT) (ND: *n* = 12; ND+Gas: *n* = 12; HFD: *n* = 15; HFD+Gas: *n* = 20); J) Area under the curve (AUC) of the ITT plot from (I). All data are expressed as mean ± SEM, three‐way ANOVA, post hoc Scheffe test (A, E–G, I), ND+Gas versus ND mice, HFD mice versus ND mice, HFD+Gas mice versus HFD mice, *p < 0.05, **p < 0.01, ***p < 0.001, NS: Not significant. 8 m‐ND versus 4 m‐ND, NS: Not significant; 8 m‐ND+Gas versus 4 m‐ND+Gas, NS: Not significant; 8 m‐HFD versus 4 m‐HFD, ***p < 0.001; 8 m‐HFD+Gas versus 4 m‐HFD+Gas, NS: Not significant, two‐way ANOVA, post hoc Scheffe test (B–D, H, J) ND+Gas versus ND mice, HFD mice versus ND mice, HFD+Gas mice versus HFD mice, *p < 0.05, **p < 0.01, ***p < 0.001.NS: not significant.

Gastrin‐SiO_2_ treatment attenuated the HFD‐induced increase in body weight (**Figure** **6**A), serum lipids (i.e., triglycerides, total cholesterol, and free fatty acids) (Figure 6B–D), and fasting and random blood glucose levels (Figure 6E,F). The gavage of Gastrin‐SiO_2_ microspheres also improved glucose tolerance (Figure 6G,H) and insulin sensitivity (Figure 6I,J). Both histopathological staining and western blotting results showed that the increased fibronectin (FN‐1), matrix metalloproteinase‐9 (MMP9), and matrix metalloproteinase‐2 (MMP2) expressions in the heart and kidney of HFD‐fed mice were normalized by the treatment with Gastrin‐SiO_2_ microspheres (Figure S4A–F, Supporting Information). Moreover, serum biochemical parameters (low‐density lipoprotein‐cholesterol [LDL‐C], creatine kinase [CK], creatine kinase myocardial band [CK‐MB], and creatinine [CR], and blood urea nitrogen [BUN]) were decreased by the treatment with Gastrin‐SiO_2_ microspheres when compared with untreated HFD mice, except for serum uric acid (UA), indicating protection of organs (such as the kidney and heart) from HFD‐induced damage (Figure S5A–F, Supporting Information). In ND‐fed mice, Gastrin‐SiO_2_ microspheres, alone, had no effect on body weight, serum lipids, blood glucose, OGTT, and ITT, or serum parameters of kidney (e.g., BUN, serum creatinine) and heart damage (e.g., serum CK‐MB) (Figure 6 and Figure S5, Supporting Information). Thus, intestinal epithelial cell‐specific stimulation of CCKBR by Gastrin‐SiO_2_ microspheres mitigated the HFD diet induced T2D phenotype in C57BL/6J mice.

### 2.5 Intestinal Gastrin/CCKBR Is a Negative Switch for SGLT1 and GLUT2 Expression

**Figure 7:**
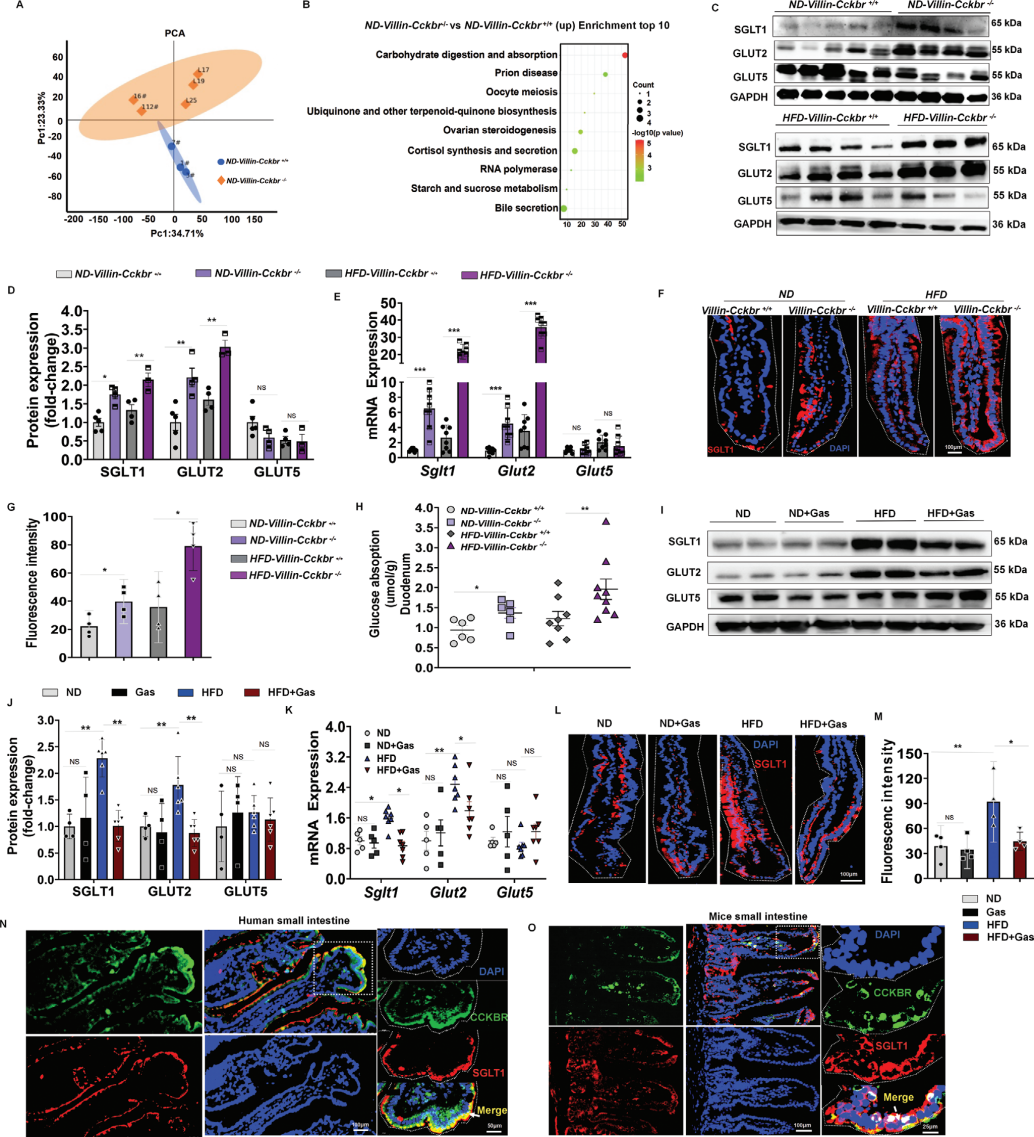
Intestinal Gastrin/CCKBR is a negative switch for SGLT1 and GLUT2 expression. A) Principal component analysis (PCA) of protein samples extracted from intestinal epithelial cell (duodenum) villi. (*ND‐Villin‐Cckbr^+/+^* mice, *n* = 3; *ND‐Villin‐Cckbr* ^−^ *^/^* ^−^mice, *n* = 5). B) Pathway mapping analysis using Kyoto Encyclopedia of Genes and Genomes (KEGG); C,D) Western blots and quantification of SGLT1, GLUT2, and GLUT5 protein expressions in the intestinal epithelial cells (duodenum) of *ND‐Villin‐Cckbr^+/+^* (*n* = 5); *ND‐Villin‐Cckbr* ^−^ *^/^* ^−^ mice (*n* = 5); *HFD‐Villin‐Cckbr^+/+^* (*n* = 5) and *HFD‐Villin‐Cckbr* ^−^ *^/^* ^−^ mice (*n* = 5). E) mRNA levels of *Sglt1, Glut2*, and *Glut5* in intestinal epithelial cells (duodenum) (*n* = 8 per group). F) Immunofluorescence of SGLT1 (red) using rabbit monoclonal antibody in the duodenum. G) Glucose uptake (absorption) in the isolated small intestines (duodenum) of the mice (*n* = 5 per group). H) Glucose absorption in isolated duodenum segment (*n* = 8–9 per group). I,J) SGLT1, GLUT2, and GLUT5 expressions and their quantification in duodenum of ND (fed with normal diet, 10% fat), ND+Gas C57BL/6J mice (ND + 20 mg kg^−1^ d^−1^ Gastrin‐SiO_2_ microspheres gavage), HFD (fed with high fat diet, 60% fat), and HFD+Gas C57BL/6J mice (fed with 60% fat diet + 20 mg kg^−1^ d^−1^ Gastrin‐SiO_2_ microspheres gavage) (*n* = 4–6 per group). K) mRNA levels of *Sglt1, Glut2*, and *Glut5* in the small intestines (duodenum) of ND, ND+Gas, HFD, and HFD+Gas C57BL/6J mice; L,M) SGLT1 immunofluorescence staining (DAPI, blue; SGLT1, red) and quantification in the small intestines (duodenum) of ND, ND+Gas, HFD, and HFD+Gas C57BL/6J mice. N,O) Immunofluorescence staining in small intestines (duodenum) from normal human and mouse (CCKBR, Green; SGLT1, Red; Yellow: merge of CCKBR and SGLT1, white arrow). All data are expressed as mean ± SEM, two‐way ANOVA, post hoc Scheffe test (D,E, G,H, J,K, M), *ND‐Villin‐Cckbr* ^−^ *^/^* ^−^ versus *ND‐Villin‐Cckbr^+/+^*, HFD‐fed *Villin‐Cckbr* ^−^ *^/^* ^−^ versus HFD‐fed *Villin‐Cckbr^+/+^* mice; HFD mice versus ND mice, HFD+Gas mice versus HFD mice, *p < 0.05, **p < 0.01, ***p < 0.001, NS: not significant.

To probe into the underlying mechanism of the protective role of Gastrin/CCKBR in T2D, a proteomic analysis of the proteins extracted from the intestinal epithelial cells was performed. The results revealed that the carbohydrate digestion and absorption pathways were upregulated after conditional small intestine‐specific *Cckbr* silencing (**Figure** **7**A,B). The protein and mRNA levels of SGLT1 and GLUT2 were increased in the ND‐fed *Villin‐Cckbr* ^−^ *^/^* ^−^ mice compared with those in the ND‐fed *Villin‐Cckbr^+/+^* mice; GLUT5 protein and mRNA expressions were not significantly changed (Figure 7C–E). SGLT1 was exclusively located in the intestinal epithelial cell membrane (Figure 7F–G). Glucose absorption (Figure 7H) in the small intestine (duodenum) in vitro was increased in the ND‐fed *Villin‐Cckbr* ^−^ *^/^* ^−^ mice relative to that in the ND‐fed *Villin‐Cckbr^+/+^* mice. HFD markedly increased the small intestinal SGLT1 and GLUT2 expressions as well as the glucose absorption in *Villin‐Cckbr* ^−^ *^/^* ^−^ mice relative to those in *Villin‐Cckbr^+/+^* mice (Figure 7C–H), whereas GLUT5 expression was not affected. In the C57BL/6J WT mice, the HFD increased the small intestinal expressions of SGLT1 and GLUT2 but not that of GLUT5, and these effects were reversed by the treatment with Gastrin‐SiO_2_ microspheres (Figure 7I–M). As shown in Figure 7N–O, immunocytochemical colocalization of SGLT1 with CCKBR was evident in human and mouse small intestines (indicated with a white arrow in the boxed area of the figure), which affirms the concept that Gastrin/CCKBR may control blood glucose levels by regulating SGLT1.

### 2.6 Gastrin/CCKBR Stimulation Decreases SGLT1 and GLUT2 Expressions by Suppressing the PI3K/Akt/eIF4B Signaling Pathway

**Figure 8:**
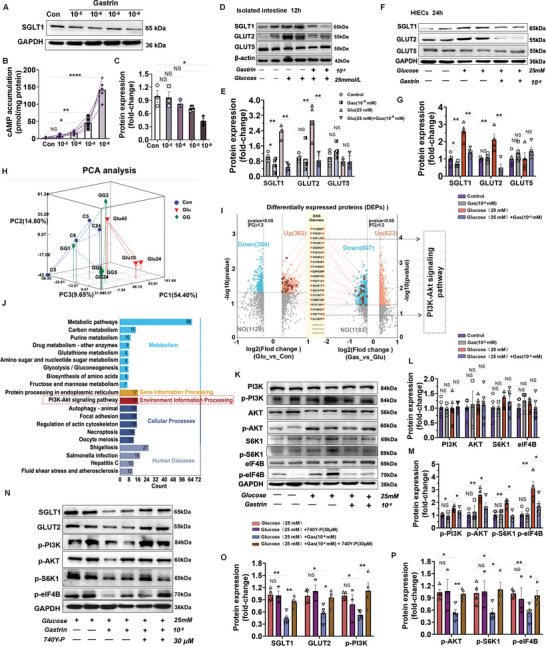
Gastrin/CCKBR stimulation suppresses PI3K/Akt/eIF4B signaling pathway to decrease SGLT1 and GLUT2 expression. A–C) Protein expression of SGLT1 and cAMP levels in HIECs incubated with different gastrin concentrations (10^−5^, 10^−6^, 10^−8^, 10^−9^ mm); D,E) Isolated duodenum from normal C57BL/6J mice were treated under different conditions as follows: control group (20 mm mannitol was added to 5 mm glucose containing medium), Glu group (25 mm glucose; Glu+Gas (25 mm glucose and 10^−9^ mm gastrin) group, *n* = 4 per group). All samples were incubated for 12 h at 37 °C and 95% O_2_ and 5% CO_2_ conditions. The proteins were extracted from the isolated HIECs and the protein levels of SGLT1, GLUT2, and GLUT5 were determined; GAPDH was used to normalize the data in isolated intestine (duodenum); F,G) Protein expression and quantification of SGLT1, GLUT2, and GLUT5; GAPDH was used to normalize the data in HIECs. H) Principal Component Analysis (PCA) analysis; I) Differentially expressed proteins (DEPs); J) KEGG enrichment analysis of the upregulated and down regulated proteins, showing the top 20‐enriched signaling pathways; K–M) Protein expression and quantification of PI3K (phosphoinositide 3‐kinase), p‐PI3K (phosphorylated‐PI3K), AKT (protein kinase B), p‐AKT (phosphorylated‐AKT), S6K1 (ribosomal protein S6 kinase β‐1), p‐S6K1 (phosphorylated‐S6K1), eIF4B (eukaryotic translation initiation factor 4B), and p‐eIF4B (phosphorylated‐eIF4B). GAPDH was used to normalize the data; N–P) Protein expression and quantification of SGLT1, GLUT2, p‐PI3K, p‐AKT, p‐S6K1, and p‐eIF4B. GAPDH was used to normalize the data. 740Y‐P (30 µm) is an activator of the PI3K/Akt signaling pathway. All data are expressed as mean ± SEM, one‐way ANOVA, post hoc Scheffe test, glucose (25 mm) versus control, gastrin (10^−9^ mm) versus control, glucose (25 mm) + gas (10^−9^ mm) versus glucose (25 m), glucose + gas + 740Y‐P (30 µm) versus glucose (25 mm) + gas (10^−9^ mm), *p < 0.05, **p < 0.01, ***p < 0.001, NS: Not significant.

Stimulation of Gastrin/CCKBR in HIECs by 10^−5^ to 10^−9^ mm gastrin decreased SGLT1 expression (**Figure** **8**A,C) and increased cAMP accumulation with a maximum effect at 10^−9^ mm (Figure 8B). In the isolated intestine and HIECs, we confirmed that a high glucose concentration (25 mm) increased the SGLT1 and GLUT2 expressions, while their expressions were decreased by gastrin treatment (10^−9 ^mm) (Figure 8D–G). By contrast, GLUT5 expression was not affected by high glucose (25 mm) treatment without or with gastrin, which is consistent with the results of the in vivo studies. To probe into the underlying mechanism of the inhibitory effect of Gastrin/CCKBR on SGLT1 and GLUT2 expression, proteomic analysis was performed in HIECs. The clustering of the samples was confirmed using Principal Component Analysis (PCA) (Figure 8H). A total of 1896 differentially expressed proteins (DEPs) were identified; 666 of these proteins were differentially expressed (362 upregulated and 304 downregulated) between the high glucose group (25 mm) and the control group, whereas 1553 DEPs were differentially expressed (623 upregulated and 607 downregulated) between the high glucose group (25 mm) and high glucose plus gastrin group (10^−9^ mm) (Figure 8I). 285 overlaps were screened using the two datasets (Glu vs Con Up and Gas vs Glu down) (Figure 8I). To gain insights into the biological pathways of the DEPs, we performed KEGG pathway analyses by using the 285 overlapping proteins. The top 20 enriched pathways are listed in Figure 8J; PI3K‐Akt signaling pathway was significantly enriched, with 17 differentially expressed genes.

We verified further the gastrin‐induced changes in the levels of the proteins related to the PI3K‐Akt signaling pathway. As shown in Figure 8K–M, phosphorylated‐PI3K (p‐PI3K, phosphorylated‐Akt (p‐AKT), phosphorylated‐S6K1 (p‐S6K1), and phosphorylated‐eIF4B (p‐eIF4B) were increased by high glucose treatment (25 mmol L^−1^), but these effects were prevented by gastrin treatment. Total PI3K, AKT, S6K1, and eIF4B expressions were not affected by glucose or gastrin treatment. Gastrin treatment, by itself, did not affect expression of these proteins, when compared to the control group (Figure S6A,B, Supporting Information). Notably, 740Y‐P, an activator of the PI3K/Akt signaling pathway,^[^ ^25^ ^]^ restored the decreased expressions of SGLT1, GLUT2, p‐PI3K, p‐AKT, p‐S6K1, and p‐eIF4B caused by gastrin in HIECs incubated in a high‐glucose (25 mmol L^−1^) medium (Figure 8N–P). Treatment with 740Y‐P, alone, did not significantly affect the protein expressions of SGLT1, GLUT2, p‐PI3K, p‐AKT, p‐S6K1, and p‐eIF4B, when compared to the high glucose group (Figure S6C,D, Supporting Information). These results suggested that Gastrin/CCKBR stimulation suppresses PI3K/Akt/eIF4B signaling pathway to decrease SGLT1 and GLUT2 expression.

### 2.7 Gastrin‐SiO_2_ Microspheres Indirectly Increase the Secretion of Incretins

**Figure 9:**
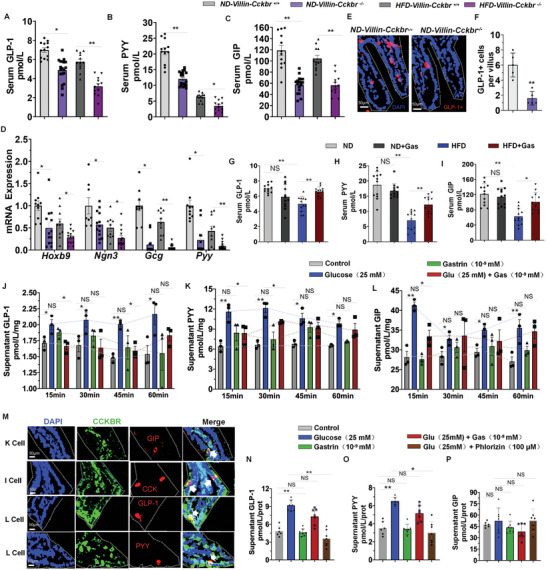
Gastrin‐SiO_2_ microspheres indirectly increase the secretion of incretins. A) Serum levels of GLP‐1 (glucagon‐like peptide 1), B) PYY (peptide YY), C) GIP (glucose‐dependent insulinotropic polypeptide) in *ND‐Villin‐Cckbr^+/+^* mice (*n* = 13) *ND‐Villin‐Cckbr^−/−^* mice (*n* = 19) mice, *HFD‐Villin‐Cckbr^+/+^* mice (*n* = 12), and *HFD‐Villin‐Cckbr^−/−^* (*n* = 12) mice; D) Expression of L cell marker genes in the small intestine (ileum); E,F) GLP‐1 (marker of L cells) immunofluorescence staining in the small intestine (ileum) in *ND‐Villin‐Cckbr^+/+^* and *ND‐Villin‐Cckbr^−/−^* mice cells (GLP‐1, Red; DAPI, Blue); G) Serum levels of GLP‐1, H) PYY, and I) GIP in C57BL/6J mice from the ND (normal diet) group (*n* = 12), ND+Gas group (*n* = 12), HFD group (*n* = 12), and HFD+Gas group (*n* = 12); J–L) Isolated mouse intestines (entire intestinal tract) were treated at different conditions for 15, 30, 45, and 60 min. The levels of gut hormones (GLP‐1, PYY, and GIP) in supernatants were measured by ELISA, and the final concentration was corrected by isolated intestine protein concentration (pmol/L/prot). As a control, 20 mm mannitol was added to 5 mm glucose containing medium, Control (5 mm glucose and 20 mm mannitol) *n* = 3, Glucose (25 mm) *n* = 3, Gastrin (10^−9 ^mm) (*n* = 3), Glucose (25 mm) + Gastrin (10^−9^ mm) *n* = 3; M) Immunofluorescence of GIP (marker of K cells, red), CCK (marker of I cells, red), and GLP‐1/PYY (marker of L cells, red) in the small intestine (ileum). CCKBR was stained green; white arrows mark the co‐localization of the marker proteins with CCKBR; N–P) Cell supernatant media were collected after 1 h treatment under the following conditions (Control, 5 mm glucose and 20 mm mannitol) *n* = 6; Glucose (25 mm), *n* = 7; Gastrin (10^−9^ mm), Glucose (25 mm) + Gastrin (10^−9^ mm), *n* = 7; 25 mm glucose+ phlorizin (100 µm), *n* = 7). The levels of gut hormones (GLP‐1, PYY, and GIP) in the supernatant were measured by ELISA, and the final concentration divided by NCL‐H716 overall protein concentration (pmol/L/prot). All data are expressed as mean ± SEM; unpaired Student's *t*‐test (F), *ND‐Villin‐Cckbr^+/+^* mice versus *ND‐Villin‐Cckbr^−/−^* mice; two way ANOVA, post hoc Scheffe test (A–D, and G–I), *ND‐Villin‐Cckbr^+/+^* mice versus *ND‐Villin‐Cckbr^−/−^* mice, *HFD‐Villin‐Cckbr^−/−^* mice versus HFD‐Villin‐Cckbr*^+/+^*, ND+Gas group versus ND group, HFD group versus ND group, HFD+Gas group versus HFD group, Glucose (25 mm) group versus Control group, Gastrin (10^−9^ mm) versus Control group, Glucose (25 mm) + Gastrin (10^−9^ mm) versus Glucose group, 25 mm glucose+ phlorizin (100 µm) versus Glucose (25 mm); three‐way ANOVA, post hoc Scheffe test (J–L), Glucose (25 mm) group versus Control group, Gastrin (10^−9^ mm) versus Control group, Glucose (25 mm) +Gastrin (10^−9^ mm) versus Glucose group, 25 mm glucose+ phlorizin (100 µm) versus Glucose (25 mm), *p < 0.05, **p < 0.01, ***p < 0.001, NS: Not significant, Control group: 60 min versus 15 min, Glucose (25 mm) group: 60 min versus 15 min, Gastrin (10^−9^ mm) group: 60 min versus 15 min, 25 mm glucose+ Gas (10^−9^ mm) group: 60 min versus 15 min, NS: Not significant.

Intestinal SGLT1 inhibition enhances the secretion of GLP‐1 in normal and diabetic rodents.^[^ ^26^ ^]^ Here, we found that the serum levels of GLP‐1, PYY, GIP, and CCK were decreased in ND‐fed *Villin‐Cckbr^−/−^* mice relative to controls (ND‐fed *Villin‐Cckbr* ^+/+^ mice) (**Figure** **9**A–C, Figure S7A, Supporting Information). In the ND‐fed *Villin‐Cckbr^−/−^* mice, there was a downregulation of the genes that positively regulate the genes expressed in L cells (*Hoxb9*, *Ngn3*, *Gcg*, and *Pyy*), I cells (*Cck*), and K cells (*Gip*) (Figure 9D, Figure S7B, Supporting Information). The expression of other gut‐specific genes, including *Muc2* (Goblet cells), *Sct* (S cells), *Dclk1* (Tuft cells), *Defa6* (Paneth cells), *Sst* (δ cells), and *Chga* (enterochromaffin cells), did not differ between the two groups (Figure S7B, Supporting Information). The abundance of L cells (GLP‐1^+^ cells) in the small intestines was also decreased in the *Villin‐Cckbr^−/−^* mice compared with that in the corresponding controls (*Villin‐Cckbr* ^+/+^ mice) under normal fat diet (Figure 9E,F). These findings were also found in the HFD‐fed *Villin‐Cckbr^−/−^* and *Villin‐Cckbr* ^+/+^ mice (Figure 9A–D, Figure S7A,B, Supporting Information).

Serum levels of GLP‐1, PYY, and GIP that were decreased in the HFD‐fed C57BL6/J mice were increased after Gastrin‐SiO_2_ microspheres administration (Figure 9G–I), while serum levels of CCK (Figure S7C, Supporting Information) were not significantly different among the four study groups. Isolated mouse intestine (duodenum) was treated as indicated at the different conditions for 15, 30, 45, 60 min; the levels of GLPs (GLP‐1, PYY, and GIP) in the supernatants of the treated small intestines were increased after high glucose (25 mm) treatment and decreased slightly with gastrin treatment (10^−9^ mm), but gastrin treatment alone had no effect on the secretion of GLPs (Figure 9J–L). Supernatant levels of CCK were not significantly different among the study groups (Figure S7D, Supporting Information).

Immunocytochemical co‐localization of CCKBR with GIP (K cells), CCK (I cells), and GLP‐1/PYY (L cells) was observed in the small intestine of the mice (Figure 9M). CCKBR was well expressed in the human enteroendocrine L cells (NCL‐H716), although the total expression was not affected by gastrin treatment (Figure S7E,F, Supporting Information). High glucose (25 mm) treatment increased the supernatant levels of GLP‐1 and PYY that were decreased by gastrin treatment (10^−9 ^mm) (Figure 9N,O) in NCI‐H716 cells. The ability of glucose to increase GLP‐1 and PYY secretion was inhibited by phlorizin (100 µm), and the supernatant levels of GIP and CCK in NCI‐H716 cells were comparable in the five group (Figure 9P, Figure S7G, Supporting Information), suggesting that glucose is crucial for GLP‐1 and PYY secretion but not gastrin stimulation, which may be influenced by the alterations in SGLT1.

To investigate the factors influencing hormone levels while avoid potential in vivo side effects, SGLT1 rescue experiments were performed ex vivo in live *Villin‐Cckbr^−/−^* mice (Figure S8A, Supporting Information). Compared to controls, intestinal tissue from *Villin‐Cckbr^−/−^* mice exposed to high glucose (25 mm) exhibited significantly increased glucose absorption in the intestinal epithelium, coupled with reduced glucose efflux into the intestinal lumen and increased secretion of intestinal hypoglycemic hormones (GLP‐1, PYY, and GIP) in both intestinal epithelial cells and luminal fluid (Figure S8B–E, Supporting Information). However, treatment with the SGLT1 inhibitor phlorizin (100 µm) largely reversed (normalized) the high glucose‐induced increase in glucose absorption, promoting glucose efflux into the lumen. Furthermore, phlorizin treatment under high glucose conditions also restored (normalized) the SGLT1‐depressed intestinal levels of GLP‐1 and PYY observed in intestinal Cckbr knockout mice, GIP also shows recovery trend. CCK secretion was not significantly affected by high glucose or phlorizin treatment (Figure S8F, Supporting Information). These data demonstrate that CCKBR negatively regulates SGLT1 expression, which, in turn, influences the levels of intestinal hormones GLP‐1, PYY, and GIP in response to changes in SGLT1 expression.

### 2.8 Gastrin‐SiO_2_ Microspheres Administration Reduces Glucose Flooding in the Primary Intestine Epithelial Cells Derived from Patients with T2D

**Figure 10:**
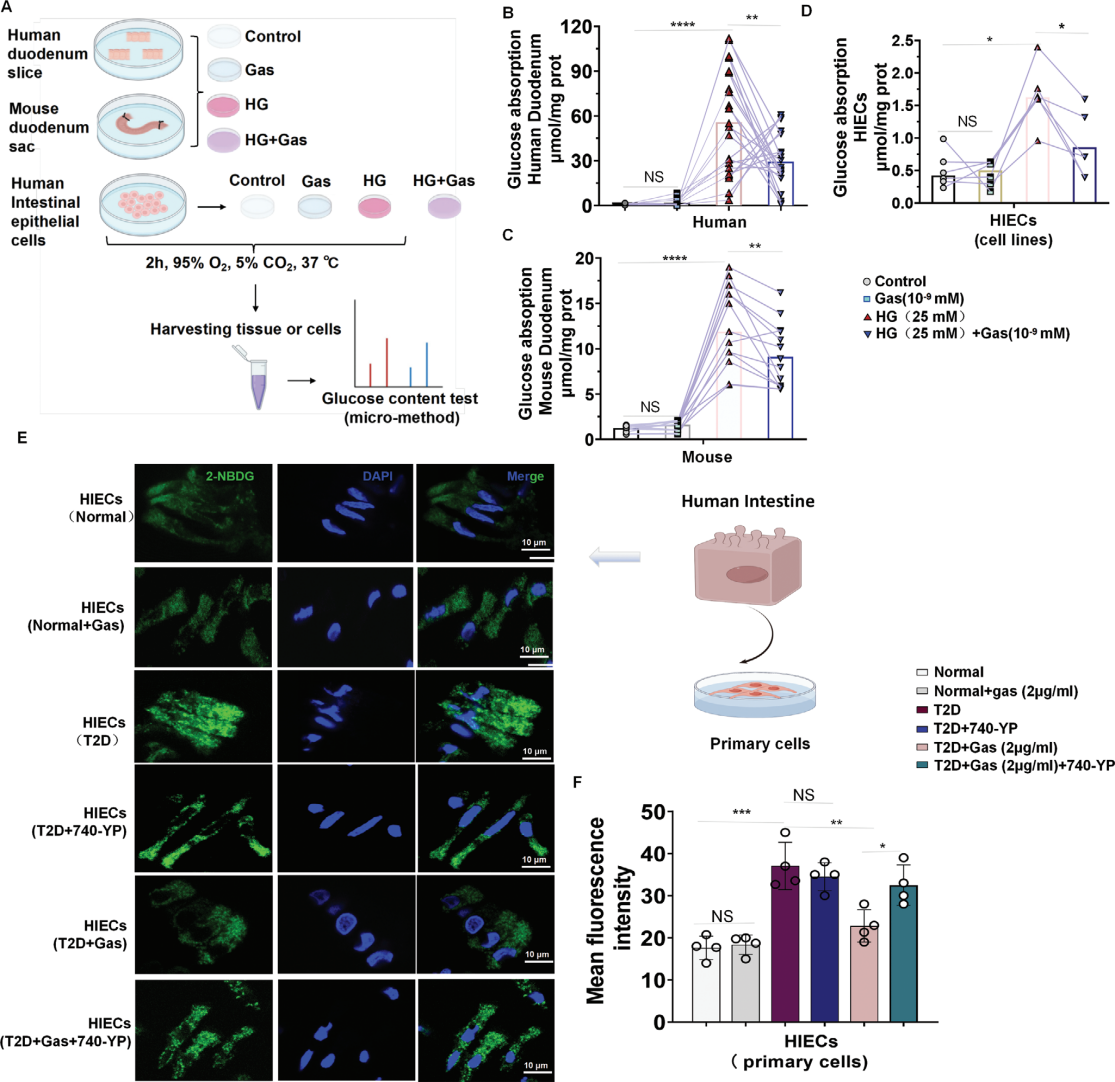
Gastrin‐SiO_2_ microspheres administration reduces glucose flooding in the primary intestine cells derived from patients with T2D. A) Flow diagram of the in vitro experiment. The effects of gastrin (10^−9^ mm) on glucose absorption in freshly isolated normal human and C57BL/6J mouse small intestines (duodenum) and glucose transport in HIECs are shown. The fresh tissues and HIECs were cultured in different media for 2 h and glucose concentrations were quantified by a colorimetric glucose uptake assay kit. B) Glucose absorption in normal human duodenum, C) normal C57BL/6J mouse duodenum, and D) HIECs. As a control, 20 mm mannitol was added to 5 mm glucose containing medium, Control (5 mm glucose and 20 mm mannitol) *n* = 5; Gastrin (Gas, 10^−9^ mm), *n* = 5; High Glucose (HG, 25 mm), *n* = 7; High Glucose + Gastrin (HD + Gas), *n* = 7; E) Immunofluorescence in primary small intestinal epithelial cells from normal persons or diabetic (T2D) patients (2‐NBDG, green; DAPI, blue) and a schematic representation of the extraction of intestinal epithelial cells; F) Mean fluorescence intensity was quantified using ImageJ software. (Normal: normal person group; Normal +gas: normal person group+Gastrin‐SiO_2_ microsphere (2 µg mL^−1^) group; T2D: type 2 diabetic patients group; T2D+740‐YP: type 2 diabetic patients+740‐YP (30 µm); DM+Gas: type 2 diabetic patients+gastrin group; T2D+Gas+740‐YP: type 2 diabetic patients+gastrin (2 µg mL^−1^)+740‐YP (30 µm), *n* = 4 per group). All data are expressed as mean ± SEM, one‐way ANOVA, post hoc Scheffe test, Glucose (25 mm) versus control, glucose (25 mm) + gas (10^−9^ mm) versus glucose (25 mm), T2D versus Normal, T2D + Gas versus T2D, T2D+740Y‐P versus T2D, T2D+Gas+740Y‐P versus T2D+Gas, *p < 0.05, **p < 0.01, ***p < 0.001, ****p < 0.0001, NS: Not significant.

In T2D, the positive imbalance of glucose intake and intestinal glucose absorption increases postprandial blood glucose levels and causes glucose metabolic dysfunction. Here we showed the effect of 10^−9^ mm gastrin on the Gastrin/CCKBR axis in freshly isolated human and mouse intestines (duodenum) and in HIECs (**Figure** **10**A). We found that gastrin was able to block or minimize the increased glucose absorption caused by high glucose (25 mm) in freshly isolated human (Figure 10B) and mouse (Figure 10C) small intestines (duodenum) and HIECs (Figure 10D).

To determine whether our therapeutic strategy in the T2D mouse model is relevant to humans, we studied the effect of Gastrin‐SiO_2_ microspheres in HIECs from healthy donors and T2D patients. The green fluorescent protein GFP fused with glucose (2(N‐(7‐Nitrobenz‐2‐oxa‐1,3‐diazol‐4‐yl)Amino)‐2‐Deoxyglucose (2‐NBDG)) was used to visualize HIEC glucose absorption. In HIECs from normal persons, Gastrin‐SiO_2_ microspheres had no effect of intracellular glucose accumulation (Figure 10E,F). However, Gastrin‐SiO_2_ microspheres were able to decrease in increased glucose accumulation in HIECs from humans with T2D (Figure 10E,F). 740Y‐P, which by itself, had no effect on increased glucose accumulation in HIECs from humans with TD, prevented the ability of Gastrin‐SiO_2_ microspheres to decrease glucose accumulation in these HIECs (Figure 10E,F). These results show that orally administered Gastrin‐SiO_2_ microspheres that specifically target the small intestine could be of great value for the clinical treatment of T2D.

## 3 Discussion

In this study, we showed the crucial role of intestinal epithelial cell Gastrin/CCKBR axis in the pathogenesis of T2D by selectively silencing and stimulating intestinal CCKBR with gavage of Gastrin‐SiO_2_ microspheres in *Villin‐Cckbr^−/−^* mice. Our studies showed four novel findings: 1) intestinal CCKBR expression is reduced in patients and mice with T2D, relative to their controls; 2) intestinal Gastrin/CCKBR axis activation normalizes the high intestinal glucose transport in *Villin‐Cckbr^−/−^* mice by inhibition of small intestine SGLT1 and GLUT2 expression through the PI3K/Akt/eIF4B signaling pathway; 3) Gastrin/CCKBR axis indirectly stimulates small intestinal secretion of incretins; and 4) oral administration of Gastrin‐SiO_2_ microspheres, by selective stimulation of the intestinal Gastrin/CCKBR axis, normalizes glucose absorption in the intestines of T2D mouse models and primary intestinal epithelial cells from T2D patients. Oral administration of gastrin‐SiO_2_ microspheres could be a promising clinical strategy for T2D treatment.

Pre‐DM, a precursor of TDM, has mild hyperglycemia which significantly increases the risk of cardiovascular diseases before the blood glucose level actually reaches the diabetic range.^[^ ^27^, ^28^ ^]^ In the current study, *Villin‐Cckbr^−/−^* mice developed a Pre‐DM phenotype at 1 month of age, indicating that the effects of *Cckbr* silencing, rather than environmental factors, contribute to diabetes progression. For individuals with Pre‐DM, lifestyle modification can reduce the risk of DM by 40–70%.^[^ ^27^ ^]^ Prolonged exposure to unhealthy diets, especially those high in carbohydrates and saturated fats, has been implicated in the pathogenesis of Pre‐DM, which progresses to overt T2D, if left uncontrolled.^[^ ^29^ ^]^ *Villin‐Cckbr^−/−^* mice developed T2D, after 1 month of HFD (60% fat)‐fed, much faster than the control mice (*Villin‐Cckbr^+/+^*) that developed T2D after 3–4 months of HFD. Moreover, these *Villin‐Cckbr^−/−^* mice already had target organ damage, e.g., collagen depositions in their kidneys and hearts, after 3–4 months of HFD. In mice, especially in C57BL/6J mice, at least 3 months are needed for HFD to induce diabetes.^[^ ^30^ ^]^ Insulin resistance, inflammation, kidney injury, and cardiac remodeling occur 6–8 months after eating a HFD.^[^ ^31^ ^]^ Our results demonstrated that intestinal epithelial cell CCKBR is a promising target in the treatment of TD, the deficiency of which significantly accelerated the progression of Pre‐DM to T2D.

In order to prove the therapeutic role of intestinal epithelial cell CCKBR in T2DM, we selectively stimulated intestinal epithelial cell CCKBR through orally administered Gastrin‐SiO_2_ microspheres in HFD‐fed mice. Oral administration of Gastrin‐SiO_2_ microspheres in these mice improved blood glucose control and decreased insulin resistance and obesity (the main hallmarks of T2D) in the HFD‐fed C57BL/6J mice. The long‐term subcutaneous infusion of gastrin protected against the hypertensive nephropathy induced by unilateral ureteral obstruction, decreased the apoptosis of the cardiomyocytes and improved the myocardial function in mice with myocardial infarction.^[^ ^32^ ^]^ However, circulating gastrin also induces cell hyper‐proliferation, and leads to cancer, including colorectal cancer.^[^ ^33^ ^]^ In the current study, we designed Gastrin‐SiO_2_ microspheres to act specifically and selectively in intestinal epithelial cells preventing the absorption of gastrin into the circulation. Inflammatory factors in the small intestines and circulating colon cancer promoting markers were not affected, demonstrating biological safety. Moreover, Gastrin‐SiO_2_ microsphere treatment conferred organ protection and delayed the development of T2D induced by a HFD diet in C57BL/6J mice.

Proteomic analysis was used to identify the mechanism(s) of the beneficial effects of intestinal epithelial cell Gastrin/CCKBR in T2D. Dietary carbohydrate has been reported to increase intestinal SGLT1 expression and activity in a concentration‐dependent manner.^[^ ^34^ ^]^ However, the KEGG pathway analysis revealed that carbohydrate digestion and absorption pathways were activated after *Gastrin/Cckbr* silencing, with a 2–3‐fold increase in SGLT1 expression and a twofold increase in GLUT2 expression. Increased intestinal SGLT1 expression allowed increased glucose transport across the apical membrane into the enterocytes, and increased GLUT2 expression allowed increased glucose absorption from inside the enterocytes into the circulation. These findings explain the development of Pre‐DM in the *Villin‐Cckbr^−/−^* mice and the HFD‐induced progression to T2D. *SGLT1* mutations, but not *GLUT2* mutations, cause major defects in intestinal glucose absorption.^[^ ^35^ ^]^ SGLT1 can stimulate the recruitment of GLUT2 to the brush border membrane of small intestines.^[^ ^36^, ^37^ ^]^ Thus, in the present study, the increase in small intestinal GLUT2 expression could have been secondary to the increased expression of SGLT1. When CCKBR was selectively stimulated in the intestine epithelial cells of mice with T2D (HFD‐induced), the increased expression of SGLT1 and GLUT2 was decreased, and the severity of diabetic phenotype was attenuated. The in vitro study in HIECs also suggested that gastrin downregulated the expression of SGLT1 and GLUT2. Therefore, activated Gastrin/CCKBR is a negative regulator of SGLT1 and GLUT2 expressions. SGLT1 inhibition has been reported to enhance GLP‐1 secretion, by reducing glucose absorption in the proximal segments of the small intestine, thereby increasing glucose delivery to distal segments of the small intestine containing endocrine cells responsive to glucose stimulation.^[^ ^38^ ^]^ Additionally, high expression of CCKBR has been observed in L cells, leading to a debate regarding the potential independent action of gastrin on GLP‐1 secretion in the distal small intestine, separate from the effects of SGLT1 blockade.^[^ ^39^, ^40^ ^]^ Our results showed that high glucose (25 mm), rather than gastrin alone, was able to stimulate the secretion of GLP‐1, PYY, and GIP in isolated small intestines and NCI‐H716 cells, suggesting an indirect action of Gastrin/CCKBR axis on intestinal endocrine cells. SGLT1 rescue ex vivo experiments were performed in live *Villin‐Cckbr^−/−^* mice to demonstrated the indirect action of Gastrin/CCKBR axis on hormone secretion.

Our study also showed that the prevention of blood glucose absorption by Gastrin‐SiO_2_ microspheres negatively regulated SGLT1, which circumvented the side effects of direct inhibition of small intestinal SGLT1. Defects in *Sglt1* lead to intestinal malabsorption of glucose and galactose in humans.^[^ ^10^ ^]^ Mice lacking *Sglt1* present with osmotic diarrhea, dehydration, and metabolic acidosis.^[^ ^13^ ^]^ T‐1095, a phlorizin analog, the first drug developed to block glucose reabsorption in the renal proximal tubule, was not considered suitable for clinical application because the inhibition of small intestinal SGLT1, led to diarrhea.^[^ ^15^ ^]^ The current study in HIECs revealed that the high glucose‐induced increase in SGLT1 expression was suppressed by the addition of gastrin. Furthermore, SGLT1 expression was only partially downregulated by the stimulation of the Gastrin/CCKBR when the glucose concentration was normal, indicating that CCKBR activation inhibited the high glucose‐mediated increase in SGLT1 expression but maintained normal SGLT1 expression under normal glucose conditions. The stool shapes and water content were normal in Gastrin‐SiO_2_ microsphere‐treated mice,^[^ ^7^ ^]^ indicating the effectiveness and bio‐safety of gastrin‐SiO_2_ microspheres, unlike T‐1095, an SGLT1 inhibitor with diarrhea as a side effect.^[^ ^15^ ^]^ Stimulation of Gastrin/CCKBR by Gastrin‐SiO_2_ microspheres directly inhibits SGLT1 expression and indirectly modulates intestinal hormone secretion, offering a potential strategy for mitigating the side effects associated with direct small intestinal administration of SGLT1 inhibitors, such as diarrhea and volume depletion,^[^ ^15^ ^]^ or systemic administration, including kidney‐related effects,^[^ ^10^, ^41^ ^]^ showing a potential therapeutic strategy for T2D.

To determine whether our findings in the T2D mouse model are relevant to humans, we studied the effect of Gastrin‐SiO_2_ microspheres in primary HIECs from healthy donors and T2D patients. We found that glucose absorption was increased in HIECs of T2D patients, relative to those from healthy donors, which was prevented by gastrin treatment. These data show that Gastrin‐SiO_2_ microspheres, administered orally, may be effective in the clinical therapeutic management of T2D.

Our current study also showed that exposure of HIECs to high‐glucose concentrations upregulated SGLT1 and GLUT2 expression and elevated p‐PI3K, p‐Akt, p‐S6K1, p‐eIF4B. Those effects were reversed by activation of Gastrin/CCKBR axis, suggesting that Gastrin/CCKBR is an upstream negative regulator of SGLT1 and GLUT2 from the perspective of protein synthesis and function. We then verified the involvement of the PI3K/Akt pathway in the inhibitory action of Gastrin/CCKBR on the high glucose‐mediated increase in the intestinal glucose transporter expression by blocking the inhibitory action of Gastrin/CCKBR on SGLT1 and GLUT2 expression, using a PI3K/Akt activator (740Y‐P). The PI3K/Akt signaling pathway is integral to cellular physiology as it facilitates growth factor signaling during organismal growth and various critical cellular processes, including glucose homeostasis, and protein synthesis.^[^ ^42^ ^]^ Recent studies suggest that targeting the PI3K/Akt signaling pathway may be a therapeutic strategy for diabetes and its associated complications.^[^ ^43^, ^44^ ^]^ Activation of this pathway has been demonstrated to enhance glucose uptake in islet and skeletal muscle cells by facilitating the translocation of glucose transporter 4 (GLUT4) from storage vesicles to the plasma membrane.^[^ ^45^ ^]^ However, the role of this pathway has rarely been reported in the intestine. Elevated glucose concentrations stimulate the PI3K/Akt signaling pathway, with subsequent activation of mTORC1, playing a pivotal role in the regulation of multiple downstream effector proteins via phosphorylation. Thus, p70 ribosomal S6 protein kinase‐1 (S6K1) and eukaryotic translation initiation factor‐4B (eIF4B), increase protein synthesis.^[^ ^46^ ^]^ The collective findings of prior research, combined with our current results, provide further support for the hypothesis that intestinal epithelial cell Gastrin/CCKBR axis stimulation inhibits, in part, SGLT1 and GLUT2 expression by down‐regulating PI3K/Akt/eIF4B signaling pathway and promoting incretins secretion.

There were several limitations in the current study. Specifically, the examination of the anti‐diabetic effects of Gastrin‐SiO_2_ microspheres was limited to T2D. However, improved glucose control and neogenesis of functional β‐cell mass have been shown with gastrin treatment,^[^ ^18^, ^19^, ^20^, ^21^, ^22^, ^23^ ^]^ suggesting that Gastrin‐SiO_2_ microspheres may also have a beneficial effect on type 1 diabetes (T1D), via the gut‐pancreas axis. Moreover, the number of samples in our human study was relatively small and therefore, the beneficial effects of Gastrin/CCKBR effects should be studied in larger cohorts of patients with T2D. Nevertheless, the targeting of intestinal Gastrin/CCKBR axis, using Gastrin‐SiO_2_ microspheres, could be a novel interventional strategy for controlling blood glucose levels in the clinical management of T2D.

## 4 Experimental Section

### Study Population

Human tissue samples were collected according to a protocol approved by the Ethics Committee of the Cancer Hospital, Chinese Academy of Medical Sciences (NCC2018B‐029). A total of 22 intestinal samples (small intestine segment, 18 healthy and four diabetic) were extracted from the OA biobank of the Cancer Hospital, Chinese Academy of Medical Sciences, in accordance with the approved guidelines. Other samples (controls) were collected from healthy individuals who died in a car accident or from those who died suddenly.

### Animals and Experimental Setup

Sex is also a biological variable. Male and female mice and patients were studied, and the findings were similar for both sexes.

*Intestine epithelial cell‐specific Cckbr knock‐out and wide‐type mice*: The loxP‐flanked *Cckbr* conditional allele (*Cckbr*‐loxP) and villin‐Cre mouse lines were generated by View Solid Biotechnology Co., LTD (Beijing, China). The loxp sites were inserted in the flanking exon 1 (from each end) of the entire *Cckbr* gene. Villin promoter Cre transgenic mice were obtained from The Jackson Laboratory (https://www.jax.org/strain/035595). Intestinal epithelial cell‐conditional *Cckbr* knockout mice (*Cckbr* ^fl/fl^ *Villin‐Cre*, cKO) were generated by breeding mice carrying two floxed *Cckbr* alleles, with mice expressing Cre from one allele of the tau locus and carrying one floxed *Cckbr* allele. The genotypes of the mice were determined by PCR of tail biopsies. Wild‐type and targeted alleles of the transgenic lines were amplified with specific primer combinations: floxed and wild‐type *Cckbr* allele: forward, 5′‐TCC TGA CTC CAA CAC CTT‐3’, and reverse, 5′‐CAG GCC TTT CTT TCC CTG GTT′; *Cckbr* null allele: forward, 5′‐TGG CAG AGT GGA GTA‐3’, and reverse, 5′‐GCC TTT CTT TCC CTG GTT‐3′; Cre *Cckbr*: forward, 5′‐GTG TGG GAC AGA GAA CAA ACG‐3’, and reverse, 5′‐TGC GAA CCT CAT CAC TCG TGC‐3’. Mice were maintained on a C57BL/6 background (Beijing HFK Bioscience Co., Ltd., Beijing, China). All the animal experiments were approved by the Institutional Animal Care and Use Committee of the Institute of Laboratory Animal Science [ILAS‐YZW19004]. Mice were handled according to the guidelines and principles published in the National Institutes of Health Guide for the Care and Use of Laboratory Animals of the National Research Council. Body weights were measured in the morning. Toe markings were used to identify mice. WT (*Cckbr* ^fl/fl^ = *Villin‐Cckbr^+/+^*) and *c*KO (*Cckbr* ^fl/fl^ *villi‐Cre* *=* *Villin‐Cckbr^−/−^*) mice of either sex were randomly divided into two groups. One group served as controls and fed normal diet (MD12031, Medicience, Jiangsu, China, 20% carbohydrate, 20% protein, 10% fat); body weight, food intake, random and fasting blood glucose levels, oral glucose tolerance test (OGTT), and insulin tolerance test (ITT) were monitored monthly for 1–8 months in *Villin‐Cckbr^+/+^* (*n* = 18) and *Villin‐Cckbr^−/−^* (*n* = 20) mice (50% female and 50% male). The other group was fed a high‐fat diet (HFD, MD12033, Medicience, Jiangsu, China; 20% carbohydrate, 20% protein, 60% fat) for 3 months, from 4 to 7 months of age (HFD‐*Villin‐Cckbr* ^+/+^ (*n* = 12, mice of either sex) or HFD‐*Villin‐Cckbr^−/−^* (*n* = 12) mice of either sex). During the treatment period, body weight, random and fasting blood glucose levels, OGTT, and ITT were monitored, as in the control group.

*HFD‐induced obesity in C57BL/6J mice*: Adult (4‐month‐old) C57BL/6J mice were purchased from Beijing HFK Bioscience Co., Ltd. (Beijing, China). Fifty mice of either sex were randomly divided into three groups: 1) Control group (fed normal diet (ND), MD12031, Medicience, Jiangsu, China, 20% carbohydrate, 20% protein, 10% fat; ND: *n* = 12, male *n* = 6, female *n* = 6); 2) HFD group (fed high fat diet for 4 months, MD12033, Medicience, Jiangsu, China, 20% carbohydrate, 20% protein, 60% fat; HFD: *n* = 15 male: female = 7:8); and 3) HFD plus Gastrin‐SiO_2_ microspheres‐gavaged group (HFD+Gastrin‐SiO_2_ microspheres gavage for 4 months, 20 mg kg^−1^ d^−1^; HFD+Gas: *n* = 25, male *n* = 13, female *n* = 12). Individual body weights, and random and fasting blood glucose levels were monitored weekly. The OGTT and ITT were monitored monthly. After 4 months of treatment, the mice were anesthetized with an intraperitoneal injection of 1.25% tribromoethanol and sacrificed. Blood, small intestine, heart, and kidneys were harvested for biochemical and histological analyses.

### Reagents and Chemicals

Gastrin‐SiO_2_ microspheres were obtained from the Beijing University of Chemical Technology. Gastrin‐1 (human, cat. no. RP12740‐0.5) were purchased from GenScript (Nanjing, China). SGLT1 (ab247121), villin (ab130751), GIP (ab25973), CCK (ab27441), CD45 (ab202220), EpCAM (ab213500), CD31 (ab281538), cAMP assay kits (ab65355) were purchased from Abcam Inc. (Cambridge, UK). Gastrin (PA5‐99460) was purchased from Thermo Fisher Scientific (Massachusetts, USA). p‐PI3K (#13857), PI3K (#4263), p‐AKT (#9271), ATK (#9272), p‐S6K1(9205), S6K1(9202) p‐eIF4B (#3591), eIF4B (#3592), and PYY (#24895) were purchased from Cell Signaling Technology, Inc. (Danvers, MA, USA). CCKBR (sc‐166690), GLUT2 (sc‐518022), GLUT5 (Sc‐271055), and GLP‐1 (sc‐57166) were purchased from Santa Cruz Biotechnology (Dallas, TX, USA). The 740Y‐P (1236188‐16‐1) was purchased from MedChem Express (New Jersey, USA). A Glucose Assay Kit (BC2505) was purchased from Solarbio Science and Technology Co., Ltd. (Beijing, China). Phlorizin was purchased from Sigma‐Aldrich (St. Louis, MO).

### Studies of Intestinal Mucosal Morphology

One cm samples of the duodenum, jejunum, and ileum were embedded in paraffin. A microtome (RM‐2235, Leica Microsystems AG., Hessen, Germany) was used to make 5 or 6 µm slices that were mounted on glass slides and subsequently stained with hematoxylin and eosin (H&E). Villus height (from the tip of the villus to the villus‐crypt junction) and crypt depth (from the villus‐crypt junction to the base of the crypt) were measured using an Olympus Van‐Ox S microscope (Opelco, Washington, DC, USA) and image analysis software (Image‐Pro, Media Cybernetics, Inc., Silver Springs, MD, USA). Six sections were taken from each intestine, and the height of the ten largest villi and the deepest crypt depth were selected from each section.

### Blood Glucose Test

*Fasting blood glucose*: The mice were fasted for 12 h (starting at 8:00 PM) before blood samples were collected for glucose measurements. Whole blood was collected from a 1 mm tail tip, and blood glucose was measured using a Bayer Contour Glucometer (Bayer, Leverkusen, Germany).

*Random blood glucose*: Random blood glucose levels were measured in the mice fed at 10:00 AM.

### Oral Glucose Tolerance Test (OGTT)

After an overnight fast, blood was collected from the tail vein 30, 60, 90, and 120 min after the mice were orally loaded with a glucose solution (0.4 g mL^−1^) at 2 g kg^−1^ body weight. Blood glucose levels were measured using a handheld glucometer (One Touch Ultra 2; LifeScan Inc., Malvern, PA, USA). An OGTT curve was drawn, and the area under the curve (AUC) was calculated for each curve.

### Insulin Tolerance test (ITT)

After a 6 h fast, ITT was performed by the intraperitoneal injection of insulin (0.75 U kg^−1^ body weight). Blood was collected by tail‐cut for glucose measurement before the injection of insulin and 30, 60, 90, and 120 min post‐insulin injection; blood glucose was measured and the area under curve was calculated. After completion of the last measurement at the final time point (120 min), a 20% glucose solution (50 mg glucose per mouse) was administered by oral gavage to mice that became hypoglycemic (blood glucose <2.77 mmol L^−1^).

### In Vitro Fluorescence Imaging

C57BL/6J mice were given saline, gastrin, PE‐Cy7‐Gastrin, or PE‐Cy7‐Gastrin‐SiO_2_ microsphere by gavage (10–20 s, 20 mg kg^−1^ d^−1^, 80 µL/10 g body weight, respectively). The mice were anesthetized with 2% isoflurane at 6, 12, 24, and 36 h after gavage, and their gastrointestinal tissues and major organs were harvested for in vitro fluorescence imaging using an IVIS Lumina III Imaging System (PerkinElmer, Boston, MA, USA). Imaging signals were analyzed using Living Image software (Xenogen, Alameda, CA, USA).

### Scanning Electron Microscopy

Mice were administered gastrin‐SiO_2_ microspheres or saline by gavage, and fecal samples (0–24 h) were collected from the mice kept in metabolic cages. The morphology of the organs (small intestine and kidneys) was examined by scanning electron microscopy (SEM). Fecal samples were mounted on aluminum specimen stubs and gold‐sputtered to 5 nm thick films to prevent beam‐charging effects (SC7640 Sputter coater, Quorum Technologies, Kent, UK). High‐resolution scanning electron microscopy was performed at 20 kV (magnification range of 30 000–120 000×) using an FEI Quanta 200 microscope (FEI, Oregon, USA), and images were processed using ImageJ software.

### Serum Analyses

Blood was harvested from non‐fasted mice to measure serum levels of GLP‐1(active) (RAB0201‐1KT, Merck Millipore, USA), PYY (RAB0413‐1KT, Merck Millipore, USA), GIP (#81511, Crystal Chem, USA), and CCK (EKE‐069‐04, Phoenix Pharmaceuticals, Germany) according to the manufacturer's protocol. For GLP‐1 measurements, plasma samples were treated with a DPP‐IV inhibitor (Merck Millipore, USA) to prevent degradation. To measure blood lipids, the mice were fasted for 12 h, blood was collected, and plasma and serum were obtained to measure serum triglyceride (A110), free fatty acid (A042), and total cholesterol (A111) levels using a chemical assay kit (Nanjing Jiancheng Bioengineering Institute).

### Immunoblotting, Immunohistochemistry, and Immunofluorescence

Immunoblotting was performed as previously described.^[^ ^25^ ^]^ Briefly, cells or tissues were lysed using cold RIPA buffer containing protease and phosphatase inhibitors (Beyotime, China). The proteins in the cell lysates were resolved using 10% SDS‐polyacrylamide gel electrophoresis at 60 V for 30 min, followed by 90 V for 2 h and then transferred electrophoretically to a 0.45 µm polyvinylidene difluoride (PVDF, Immobilon‐P; Millipore, Billerica, MA) membranes at 100 V for 60 min. The membranes were then incubated for 1 h at room temperature with blocking buffer (40 mL PBS, 0.1% Tween‐20, 2 g of Marvel Dried Milk Powder), followed by an overnight incubation with the appropriate primary antibody (see below) at 4 °C. The membranes were washed 3 times for 7 min each with washing buffer (PBS, 0.1% Tween 20), followed by incubation with the appropriate secondary antibody: anti‐mouse IgG (ab6728, Abcam, USA), anti‐rat IgG (ab6734, Abcam, USA), or anti‐rabbit IgG (ab6721, Abcam, USA) for 1 h at room temperature. Western blot signals were measured by chemiluminescence using the Immobilon Western Chemiluminescence HRP substrate (Millipore, WBKLS0500). The primary antibodies were used as previously described. The uniformity of protein loading and membrane transfer was determined by immunoblotting for GAPDH.

*Immunohistochemistry and immunofluorescence* studies were performed in 5 µm thick sections, obtained using microtome (Leica RM2235; Leica Microsystems), of mouse small intestines and kidneys fixed in 10% formalin (pH of 7.4), as previously reported.^[^ ^25^ ^]^ The primary antibody used in the immunochemistry studies was against SGLT1 (203‐1‐AP, 1:100, Proteintech), whereas the primary antibodies used in the immunofluorescence studies were against villin, CCK, CD31, CD45, EpCAM, gastrin, GIP, GLP‐1, PYY, and SGLT1. DAPI was used to stain the nuclei. Immunofluorescence was observed using fluorescence or confocal fluorescence microscopy (Leica Microsystems GmbH, Wetzlar, Germany).

### Quantitative Reverse‐Trasncription Polymerase Chain Reaction

Total RNAs of the small intestines and cells was purified using TRIzol (Invitrogen), following the manufacturer's instructions and as previously reported.^[^ ^7^ ^]^ The RNA samples were converted into first‐strand cDNA using PrimeScript Reverse Transcriptase (036A, Takara, Dalian, China), following the manufacturer's protocol. Quantitative gene expression was measured by real‐time quantitative polymerase chain reaction (q‐PCR) using ABI Prism 7900 HT (Applied Biosystems, Foster City, CA, USA) and the SYBR Green real‐time PCR detection method (820A, Takara, Dalian, China). The primers used are listed in Table S1 (Supporting Information). The DNA expressions were normalized by GAPDH and quantified using 2‐ΔΔCt. Primers for the small intestine glucose transport genes and enteroendocrine cell subset‐specific genes are shown in Table S1 (Supporting Information).

### Hematoxylin and Eosin (H&E) Staining

Freshly obtained small intestines from *Villin‐Cckbr^+/+^* and *Villin‐Cckbr^−/−^* mice fed normal or HFD and WT, WT+HFD, and WT+HFD+Gas mice were immediately fixed in 4% paraformaldehyde solution for 48 h, after which time the samples were embedded in paraffin and cut into 4 µm‐thick slices. The tissues were stained with H&E and observed under a light microscope (TI‐S, Nikon, Japan). Images were collected and analyzed using NDP view 2 (Hamamatsu software).

### Measurement of Glucose Absorption in Isolated Mouse and Human Duodenum

Krebs buffer was prepared (118 mm NaCl, 5 mm KCl, 1.328 mm CaCl_2_, 1.2 mm KH_2_PO_4_, 1.2 mm MgSO_4_, and 25 mm NaHCO_3_). The ligated small intestinal sac of mice was used as an ex vivo model to study the effects of gastrin (10^−9^ mm) on intestinal absorption. Krebs buffer with or without 25 mm glucose or 25 mm glucose plus gastrin (10^−9^ mm) was infused into the open end of the freshly obtained mouse small intestine, which was immediately ligated at the other end. The gut sac was then placed in Krebs buffer and cultured for 2 h at 37 °C in an incubator with 5% CO_2_ and 95% oxygen (Steri‐Cult CO_2_ Incubator, Labotec, South Africa). Krebs buffer and Krebs buffer with glucose, but without gastrin, were used as controls. Glucose concentrations were measured in all incubation solutions (inside and outside the intestinal sac) before and after the 2 h incubation period, and the sugar in these small intestines was identified using a commercial assay kit (BC2505, Solarbio, China). The intestinal glucose absorption was calculated as the amount (mg) of glucose absorbed per gram of mouse duodenum using the following formula: Glucose content (µmol g^−1^ mass) = (*C* standard × *V*1) × (*A*3–*A*1) ÷ (*A*2–*A*1) ÷ (*W* × *V*1 ÷ *V*2) = 2 × (*A*3–*A*1)(*A*2–*A*1) ÷ *W* (*C*: standard concentration in standard tube (2 µmol mL^−1^); *V*1: volume of sample added (0.02 mL); *V*2: total volume of sample added (1 mL); *A*1: absorbance of blank; *A*2: standard pipe of absorbance; *A*3: absorbance of the determination tube; *W*: sample weight (g).

Human duodenal samples were obtained with informed consent and approved by the Ethics Committee of the Cancer Hospital, Chinese Academy of Medical Sciences (NCC2018B‐029). The human duodenum was cut open longitudinally, cut into ≈5 mm pieces, extensively washed with cold Krebs buffer, and then incubated in Krebs buffer, Krebs buffer with glucose (25 mm), and Krebs buffer with glucose (25 mm) plus gastrin (10^−9^ mm) for 2 h, as described above. Duodenal glucose concentrations were measured using a commercial assay kit (BC2505, Solarbio, China). Duodenal glucose absorption was calculated as the amount (mg) of glucose absorbed per mg protein of human duodenum, using the following formula: Glucose content (µmol mg^−1^ prot) = (*C* standard × *V*1) × (*A*3–*A*1) ÷ (*A*2–*A*1) ÷ (*V*1 × Cpr) = 2 × (*A*3–*A*1) ÷ (*A*2–*A*1) ÷ Cpr (Cpr: sample protein concentration (mg mL^−1^)).

### In Situ Intestine Perfusion Studies

Prior to experimentation, the *Villin‐Cckbr^−/−^* mice were subjected to an overnight fasting period of ≈16 h, during which they had unrestricted access to water. Anesthesia was induced by an intraperitoneal injection of 2.5% tribromoethanol. Subsequently, mice were placed on a warming pad to maintain their body temperature. The abdominal region was sterilized using isopropyl alcohol, and a 1.5‐cm midline incision was made longitudinally to expose and isolate the small intestine, ≈2 cm distal to the ligament of Treitz. Incisions were made at both the proximal and distal ends. Glass cannulas with an outer diameter of 2.0 mm and connected to flexible polyvinyl chloride tubing were inserted at both ends of the small intestine and secured using silk sutures. The inlet tubing was attached to a 20‐mL syringe mounted on a perfusion pump (Model 22; Harvard Apparatus, South Natick, MA, USA), while the outlet tubing was directed into a collection vial. The perfusion buffer, with a pH of 6.5, comprised 10 mm MES, 135 mm NaCl, 5 mm KCl, and 100 µm phlorizin, with or without the addition of 25 mm high glucose, achieving an osmolarity of 290 mOsm L^−1^. This buffer was equilibrated at 37 °C to simulate physiological body temperature and subsequently perfused through the small intestine at a flow rate of 0.1 mL min^−1^ for 30 min, after which the proximal intestine was collected to assess glucose absorption and intestinal luminal fluid was obtained to quantify the levels of GLP‐1, PYY, GIP, and CCK. Following this procedure, the protein concentration of the perfused intestinal segment was precisely measured to normalize the aforementioned data.

### Isolation of Intestinal Epithelial Cells (IECs)

To confirm the deletion of *Cckbr* in intestinal epithelial cells of *Villin‐Cckbr^−/−^* mice, small intestines were isolated, cut‐opened longitudinally, and rinsed with pre‐chilled PBS. To disrupt epithelial integrity, the small intestine was placed in 1 mm dithiothreitol (DTT) for 30 min at 37 °C on a shaker and then vortexed for 1 min. The liberated IECs were collected, resuspended in 5 mL of 20% Percoll, and then overlaid with 40% Percoll in a 15 mL Falcon tube. Percoll gradient separation was performed by centrifugation at 780*g* for 20 min at 25 °C. The interface cells were collected and used as the IECs.^[^ ^45^ ^]^ Human primary IECs were isolated from fresh intestinal samples as described above.

### Glucose Uptake in Primary Human Intestinal Epithelial Cells (HIECs)

Primary HIECs, immediately prepared or after overnight attachment, were treated for 2 h with or without gastrin (10^−9^ mm) when glucose uptake was measured using 2‐NBDG. The cells were incubated with 10 mm 2‐NBDG in PBS for 15 min at 37 °C and extracted using a lysis buffer. The cells were washed five times with PBS, and a Nikon Eclipse Ti‐s microscope (Nikon) was used to observe the fluorescence. The mean fluorescence intensity was measured using the ImageJ software.

### Cell Culture

HIECs and NCI‐H716 cells were obtained from the Cell Bank of Chinese Academy of Sciences (Shanghai, China). HIECs were cultured in high glucose DMEM (4500 mg L^−1^), and NCI‐H716 cells were cultured in RPMI 1640 medium (GIBCO, Thermo Fisher Scientific, Waltham, MA, USA). All media were supplemented with 10% fetal bovine serum, 100 units mL^−1^ penicillin, and 100 µg mL^−1^ streptomycin. HIECs were cultured under several experimental conditions, i.e., normal glucose (5 mm), high glucose (25 mm), high glucose + gastrin (10^−9^ mm), and high glucose + gastrin (10^−9^ mm) + 740Y‐P (30 µm), betulinic acid, an activator of PI3K. After 24 h of treatment, the cells were collected and used for subsequent experiments. NCI‐H716 cells were treated with normal glucose (5 mm), high glucose (25 mm), gastrin (10^−9^ m), or high glucose + gastrin (10^−9^ mm). After treatment for 24 h, cell supernatants were collected to measure the GLP‐1, PYY, GIP, and CCK levels. For the control, 20 mm mannitol was added to 5 mm glucose‐containing medium for both HIECs and NCI‐H716 cells.

### ITRAQ‐Based Quantitative Proteomics

Protein samples were obtained from the small intestines of *Villin‐Cckbr^+/+^* and *Villin‐Cckbr^−/−^* mice or HIECs and stored at −80 °C in a freezer until use. iTRAQ‐based quantitative proteomic and bioinformatics analyses were performed as previously described.^[^ ^46^ ^]^

### Data Analyses

All data are expressed as mean ± SEM (standard error of the mean). All statistical analyses were performed using the Prism software (GraphPad). The difference between the two groups was analyzed using Student's *t*‐test or one‐way or two‐way analysis of variance (ANOVA), followed by the Scheffé’s test, including all time points in the two groups. Differences among three or more groups were analyzed by one‐way, two‐way, or three‐way ANOVA, followed by Scheffé’s test. Statistical significance was set at *p* < 0.05.

## Conflict of Interest

The authors declare no conflict of interest.

## Author Contributions

X.L. designed the research studies, conducted the experiments, analyzed the data, and wrote the manuscript. X.L., Y.P.L., and AX.L. contributed to data analysis and provided the reagents. W.L. and S.Y.S. contributed to the performance of the animal experiments. S.B.L., X.X.W., and X.D.J. contributed to the collection of patient samples. P.A.J. contributed to the manuscript review. Q.W. performed the experiments and animal data analyses. X.L.J. and H.Z.Z. contributed to the experimental design and manuscript review and provided financial support for the research. Z.W.Y. is the guarantor of this work and, as such, provided financial support for the research, had full access to all the data in the study, and takes responsibility for the integrity of the data and the accuracy of the data analysis. All authors approved the final version of the manuscript.

## Supporting information



Supporting Information

## Data Availability

The data that support the findings of this study are available in the Supporting Information of this article.
