# Supplementary material for: Intestinal Gastrin/CCKBR Axis Protects against Type 2 Diabetes by Reducing Intestinal Glucose Absorption through the PI3K/Akt/eIF4B Signaling Pathway
Source: Adv Sci (Weinh). 2025 Feb 14;12(13):2410032. doi: 10.1002/advs.202410032 (PMC11967862; doi:10.1002/advs.202410032)
Supplement: Supplementary file 1 — Supporting Information [file ADVS-12-2410032-s001.docx]

## Supplementary Materials

## Intestinal Gastrin/CCKBR axis Protects Against type 2 Diabetes by Reducing Glucose Absorption through the PI3K/Akt/eIF4B Signaling Pathway

Xue Liu^1,2^, Xing Liu^1^, Yunpeng Liu^1^, Anxiong Long^1^,Wei Liu^3^, Shiyun Sun^1^, Shuaibing Lu^4,5^, Xianxian Wu^1^, Xiaodi Jia^6^, Pedro A Jose^7,8^, Qiang Wei^1^, Xiaoliang Jiang^1*^, Haizeng Zhang^4,5*^, Zhiwei Yang^1*^

^1^Institute of Laboratory Animal Sciences (CAMS & PUMC), National Center of Technology Innovation for Animal Model，National Human Diseases Animal Model Resource Center, NHC Key Laboratory of Human Disease Comparative Medicine, Beijing Engineering Research Center for Experimental Animal Models of Human Critical Diseases, Beijing 100021, P.R. China.

^2^Department of Cardiology, the Second Affiliated Hospital, School of Medicine, Zhejiang University, State Key Laboratory of Transvascular Implantation Devices, Heart Regeneration and Repair Key Laboratory Zhejiang Province, Hangzhou, 310009, China.

^3^Graduate School of Hebei North University, Zhangjiakou 075031, Hebei, China.

^4^Department of Colorectal Surgery, National Cancer Center/National Clinical Research Center for Cancer/Cancer Hospital, Chinese Academy of Medical Sciences and Peking Union Medical College, Beijing, 100021, China.

^5^State Key Laboratory of Molecular Oncology, National Cancer Center/National Clinical Research Center for Cancer/ Cancer Hospital, Chinese Academy of Medical Sciences and Peking Union Medical College, Beijing, 100021, China

^6^Taihe County People's Hospital, The Taihe Hospital of Wannan Medical College. 21 Jiankang Road, Taihe County, Anhui Province, 236600, P.R. China.

^7^Department of Pharmacology and Physiology, The George Washington University School of Medicine & Health Sciences, Washington, DC, 20052, USA.

^8^Department of Medicine, Division of Kidney Diseases & Hypertension, The George Washington University School of Medicine & Health Sciences, Washington, DC, 20052, USA.

*** Corresponding author**

* Xiaoliang Jiang, PhD, Associate Professor of Physiology

Institute of Laboratory Animal Sciences(CAMS & PUMC), National Center of Technology Innovation for Animal Model, National Human Diseases Animal Model Resource Center, NHC Key Laboratory of Human Disease Comparative Medicine, Beijing Engineering Research Center for Experimental Animal Models of Human Critical Diseases, 5 Pan Jia Yuan Nan Li Chaoyang District, Beijing 100021, P.R. China

Email: [rinoa2007@126.com](mailto:rinoa2007@126.com)

* Haizeng Zhang, MS, Professor of Colorectal Surgery

Department of Colorectal Surgery, National Cancer Center/National Clinical Research Center for Cancer/Cancer Hospital, Chinese Academy of Medical Sciences and Peking Union Medical College, Beijing, 100021, China.

Email: [haizengzhang@163.com](mailto:haizengzhang@163.com)

* Zhiwei Yang , MD, Professor of Physiology,

Institute of Laboratory Animal Sciences(CAMS & PUMC), National Center of Technology Innovation for Animal Model, National Human Diseases Animal Model Resource Center, NHC Key Laboratory of Human Disease Comparative Medicine, Beijing Engineering Research Center for Experimental Animal Models of Human Critical Diseases,5 Pan Jia Yuan Nan Li Chaoyang District, Beijing 100021, P.R. China

Tel: 0086-10-67776809 Fax: 0086-10-67770685

Email: [yangzhiwei@cnilas.pumc.edu.cn](mailto:yangzhiwei@cnilas.pumc.edu.cn)

**Supplementary tables**

|  | **Primer (F)** | **Primer (R)** |
| --- | --- | --- |
| *Sglt1* | GTGGTACCGTTGGAGGCTT | CCACAAAGTGACCACTTCCA |
| *Glut2* | TATAGACATGTTTTGGGTGTTCCAC | AGGCCTGAAATTAGCCCTTC |
| *Glut5* | AGGCTTCTCACAGTCTCCCA | TGCCGCTCACCTCCTTTTAG |
| *Hoxb9* | TCTGGGACGCTTAGCAGCTAT | GCCCGAAGGAAACTTGGCT |
| *Gcg* | TGTCTACACCTGTTCGCAGC | TCCTCTGTGTCTTGAAGGGC |
| *Ngn3* | GTCACTGACTGACCTGCTGC | AGGTTGTTGTGTCTCTGGGG |
| *Pyy* | CCTCCTGCTCATCTTGCTTC | CAGGATTAGCAGCATTGCGA |
| *Cck* | ACTGCTAGCGCGATACATCC | TTATTCTATGGCTGGGGTCC |
| *Gip* | GTACGCGGAAGGGACTTTCA | TTCTTGGCTGGGGAGCTCTG |
| *Pax6* | CGGGACTTCAGTACCAGGG | CTTCATCCGAGTCTTCTCCG |
| *Muc2* | ATGCCCACCTCCTCAAAGAC | GTAGTTTCCGTTGGAACAGTGAA |
| *Sct* | CAGGGTCTGGTGGGGAAG | GAGCTGGTCCTCTAAGGGCT |
| *Dclk1* | TGAAGCGCCTGTACACTCTG | CTTCTCTGGTCCACATGCAA |
| *Chga* | ATGACAAAAGGGGACACCAA | GTCTCCAGACACTCAGGGCT |
| *Defa6* | GGACCAGGCTGTGTCTGTCT | TTGCAGCCTCTTGCTCTACA |
| *Sst* | CAGACTCCGTCAGTTTCTGC | TTCTCTGTCTGGTTGGGCTC |

**Table S1**: Primers for small intestine glucose transport genes and enteroendocrine cell subset-specific genes.

|  | **2 mo-old** | **4 mo-old** | **6 mo-old** | **8 mo-old** |
| --- | --- | --- | --- | --- |
| **Food intake (g/day)** | | | | |
| ***Villin-Cckbr ^+/+^*** | 3.43±0.72 | 4.70±0.31 | 5.12±0.0.32 | 5.74±0.44 |
| ***Villin-Cckbr ^-/-^*** | 3.56±0.67 | 4.63±0.17 | 5.48±0.44 | 5.83±0.37 |
|  | p=0.5492 | p=0.7496 | p=0.1187 | p=0.3368 |

**Table S2**: **Food intake in *Villin-Cckbr^+/+^* mice and *Villin-Cckbr^-/-^* mice.** Food intake was calculated in group-housed mice by averaging the food intake for the cage, divided by the number of mice in the cage. All data are expressed as mean ± SEM, two way ANOVA, post hoc Scheffe test, *Villin-Cckbr^+/+^* mice (n=18) vs *Villin-Cckbr^-/-^* mice (n=20).

|  | **5 mo-old** | **6 mo-old** | **7 mo-old** |
| --- | --- | --- | --- |
| **Food intake (g/day)** | | | |
| **HFD-*Villin-Cckbr^+/+^*** | 5.72±0.12 | 5.67±0.11 | 5.75±0.10 |
| **HFD-*Villin-Cckbr^-/-^*** | 5.16±0.45 | 5.57±0.52 | 5.91±0.46 |
|  | p=0.3326 | p=0.7275 | p=0.2873 |

**Table S3: Food intake in HFD-fed *Villin-Cckbr^+/+^* mice and *Villin-Cckbr^-/-^* mice.** Mice at 4 months of age were fed HFD for 3 months. All data are expressed as mean ± SEM, two way ANOVA, post hoc Scheffe test, *Villin-Cckbr^+/+^* mice (n=12) vs *Villin-Cckbr^-/-^* mice (n=12).

|  | **5 mo-old** | **6 mo-old** | **7 mo-old** | **8 mo-old** |
| --- | --- | --- | --- | --- |
| **Food intake (g/day)** | | | |  |
| **ND** | 5.63±0.09 | 5.08±0.49 | 4.89±0.31 | 4.90±0.44 |
| **NG+Gas** | 5.67±0.08 | 5.034±0.11 | 4.81±0.25 | 4.89±0.51 |
| **HFD** | 5.77±0.25 | 5.78±0.23 | 5.17±0.72 | 5.59±0.84 |
| **HFD+Gas** | 5.71±0.37 | 4.94±0.32 | 4.93±0.37 | 7.07±0.26 |
| **Control vs HFD** | p=0.5698 | p=0.5219 | p=0.6287 | p=0.2916 |
| **HFD vs HFD+Gas** | p=0.7414 | p=0.1019 | p=0.3102 | p=0.1034 |

**Table S4: Food intake in C57BL/6J mice fed normal diet or high fat diet (HFD) with or without Gastrin-SiO_2_ (Gas) microspheres** . Mice at 4 months of age were fed normal diet (ND, n=12), high fat diet (HFD, n=15), or high fat diet and gavaged with Gastrin-SiO_2_ microspheres (HFD+Gas, n=25) for 4 months. All data are expressed as mean ± SEM, three-way ANOVA, post hoc Scheffe test, HFD mice vs Control mice, HFD+Gas mice vs HFD mice.

**Supplementary figures and figure legends**

**Figure S1**

**
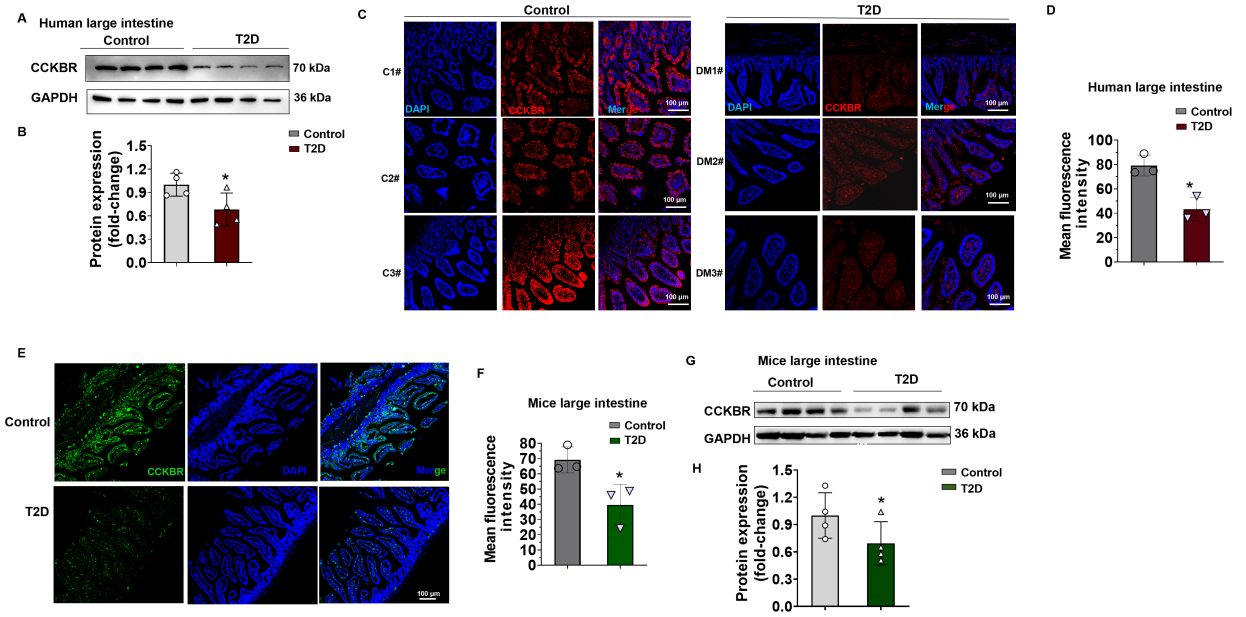
**

**Figure S1: Large intestinal CCKBR expression is decreased in patients and mice with T2D. A-B:** Western blots and quantification of CCKBR in the large intestine (colon) of normal humans (control) and T2D patients (n=4/group). **C-D:** Immunofluorescence and quantification of CCKBR in the large intestine (colon) in normal humans (control) and T2D patients (CCKBR, red; DAPI, blue, n=3/group). **E-F:** Immunofluorescence and quantification of CCKBR in the large intestine (colon) of control mice and HFD-induced T2D mice, (CCKBR, green; DAPI, blue, n=3/group). **G-H**: Western blots and quantification of CCKBR in the large intestine (colon) of control mice and HFD-induced T2D mice (n=4/group). All data are expressed as mean ± SEM. Unpaired Student’s t-test, Control vs T2D, *P<0.05.

**
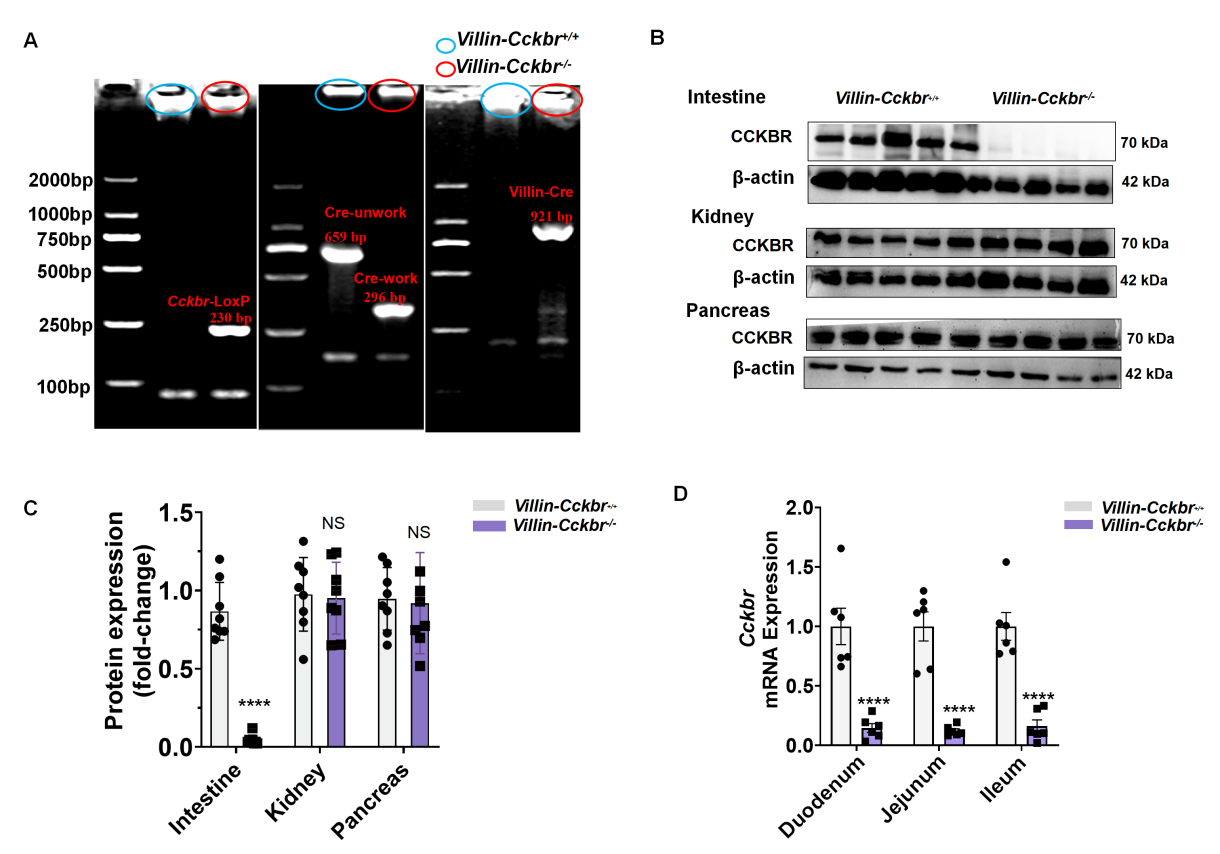
**

**Figure S2: Characterization of intestinal epithelial cell *Cckbr* conditional knockout mice.** **A:** DNA fragmentation was detected by agarose gel electrophoresis. Cckbr-Loxp (230 bp), Cre-work (276 bp), and villi-cre (921 bp) were detected in *Villin-Cckbr^+/+^* and *Villin-Cckbr^-/-^* mice; **B-C:** Western blots and quantification of CCKBR in intestine epithelial cells (isolated from the duodenum), kidney, and pancreas in *Villin-Cckbr^+/+^* (n=8) and *Villin-Cckbr^-/-^* (n=8) mice; **D**: mRNA levels of *Cckbr* in villi of small intestine epithelial cells (isolated from duodenum, jejunum, and ileum) in *Villin-Cckbr^+/+^* (n=6) and *Villin-Cckbr^-/-^* (n=6) mice. All data are expressed as mean ± SEM. unpaired Student’s t‐test, *Villin-Cckbr*^+/+^ mice vs *Villin-Cckbr^-/-^* mice, ****P<0.0001.


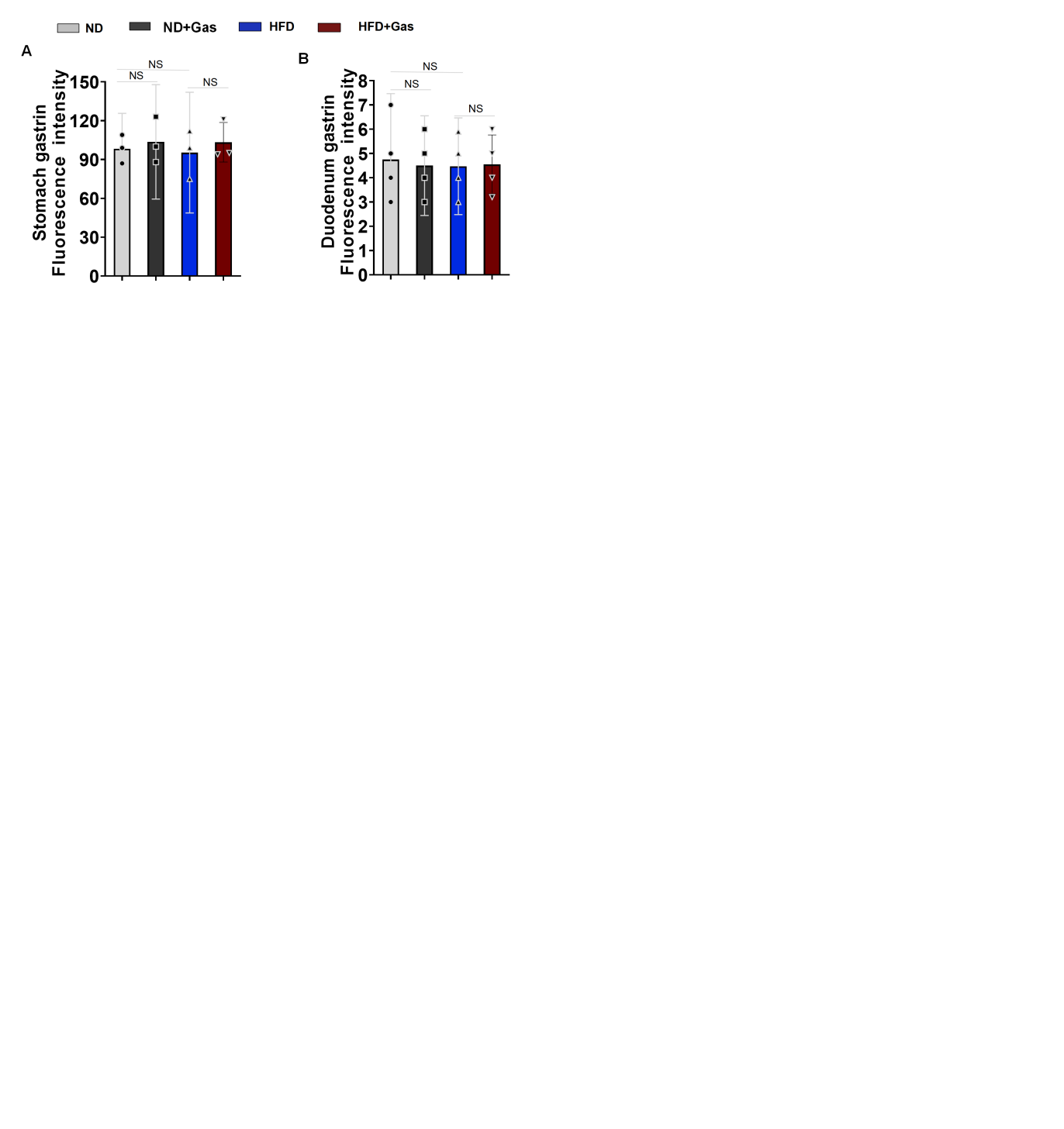


**Figure S3: Immunofluorescence of gastrin in stomach and intestine.** Immunofluorescence of gastrin (Gas) in stomach (**A**) and small intestine (duodenum) (**B**), n=3/group. Fluorescence quantified by Image J. All data are expressed as mean ± SEM, two-way ANOVA, post hoc Scheffe test, HFD mice vs ND (normal diet) mice, HFD+Gas mice vs HFD mice. NS = not significant

**
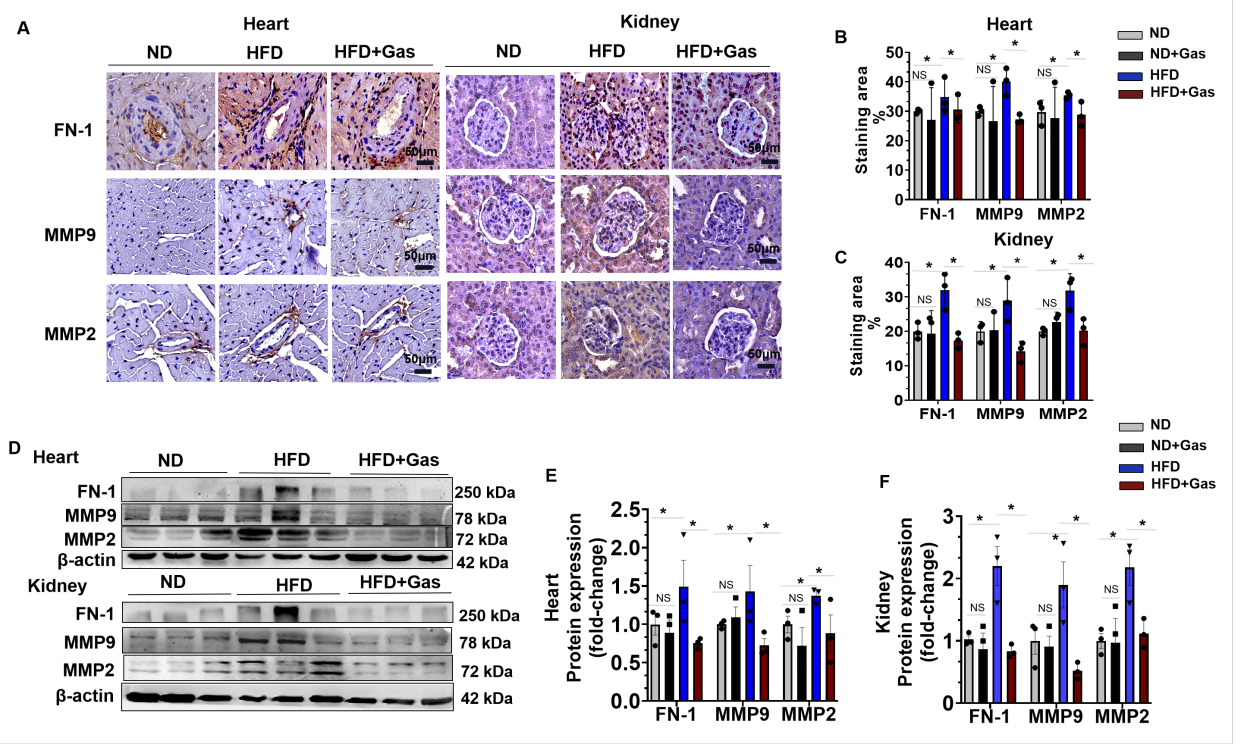
Figure S4: Stimulation of intestinal epithelial cell Gastrin/CCKBR decreases collagen deposition in HFD-fed C57BL/6J mice. A-C:** Immunohistochemistry of FN-1, MMP9, and MMP2 proteins in the heart and kidney (×400) of ND, ND+Gas, HFD, and HFD+Gas mice. Quantification of the immunohistochemical analysis was performed using ImageJ software, n=3/group; **D-F**: Heart and kidney fibronectin (FN-1) and matrix metallopeptidases (MMP9, and MMP2) proteins, quantified by western blot (n=3/group). All data are expressed as mean ± SEM, two-way ANOVA, post hoc Scheffe test, HFD mice vs ND mice, HFD+Gas mice vs HFD mice, ND+Gas vs ND, *P<0.05, **P<0.01, NS = not significant

**
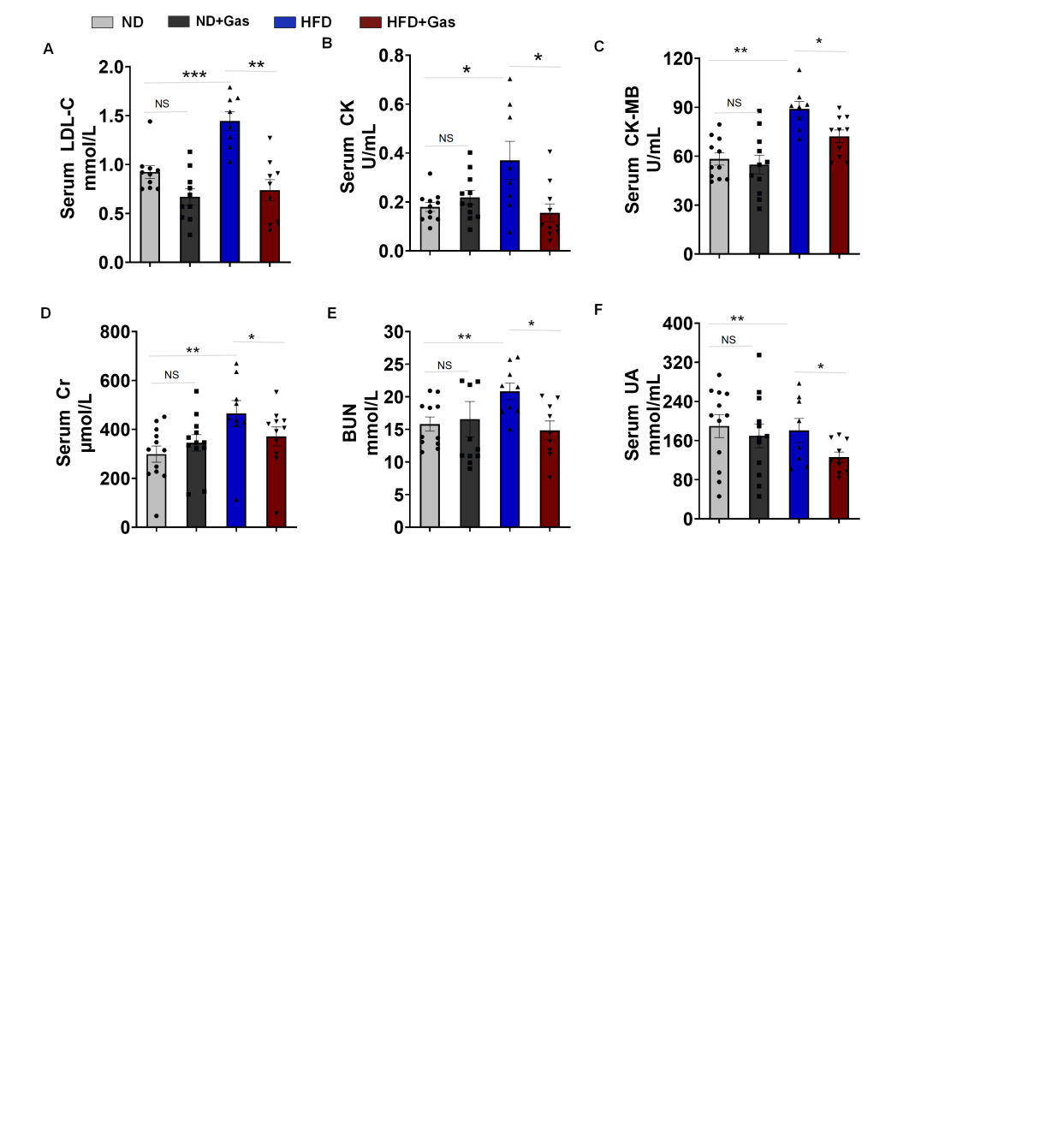
**

**Figure S5: Stimulation of intestinal epithelial cell Gastrin/CCKBR decreases the target organ damage in HFD-fed C57BL/6J.** Serum levels of low-density lipoprotein cholesterol (LDL-C) (**A**), creatine kinase (CK) (**B**), creatine kinase myocardial band (CK-MB) (**C**), serum creatinine (Cr) (**D**), blood urea nitrogen (BUN) (**E**), and serum uric acid (UA) (**F)** (n=8-15/group). All data are expressed as mean ± SEM, two-way ANOVA, post hoc Scheffe test, HFD mice vs ND mice, HFD+Gas mice vs HFD mice, ND+Gas vs ND, *P<0.05, **P<0.01, ***P<0.001, NS = not significant.

**
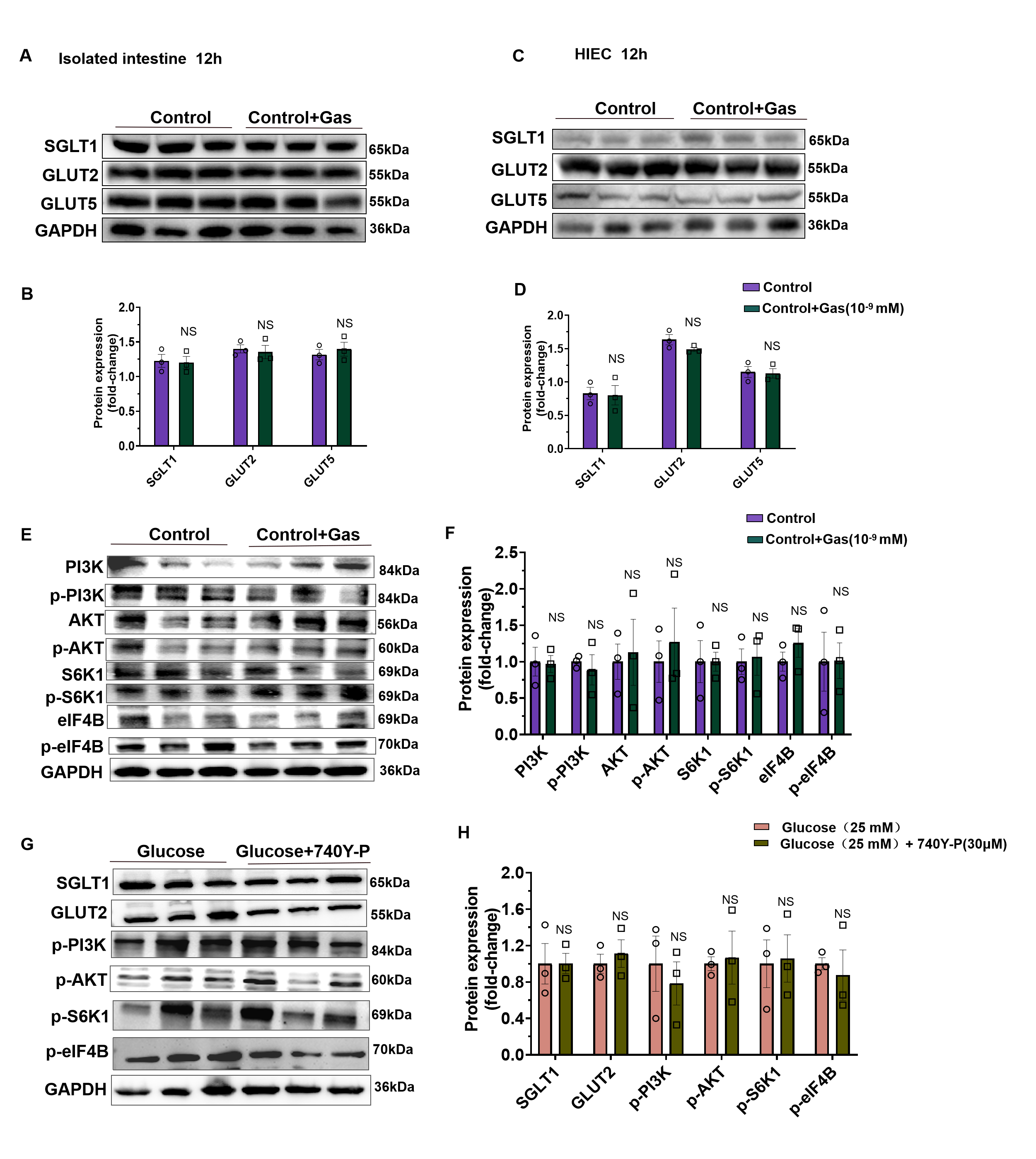
Figure S6: SGLT1 expression in control groups and PI3K/Akt/eIF4B signaling pathway expression in HIECs treated with gastrin or 740Y-P**. **A-B**: Protein expression and quantification of SGLT1, GLUT2, and GLUT5 in Isolated duodenum from normal C57BL/6J mice; GAPDH was used to normalize the data in isolated intestine (duodenum); **C-D**: Protein expression and quantification of SGLT1, GLUT2, and GLUT5; GAPDH was used to normalize the data in HIECs; **E-F:** Protein expressions and quantification of PI3K (phosphoinositide 3-kinase), p-PI3K (phosphorylated-PI3K), AKT (protein kinase B), p-AKT (phosphorylated-AKT), S6K1 (ribosomal protein S6 kinase b-1), p-S6K1 (phosphorylated-S6K1), eIF4B (eukaryotic translation initiation factor 4B), and p-eIF4B (phosphorylated-eIF4B). GAPDH was used to normalize the data. **G-H:** Protein expressions and quantification of SGLT1, GLUT2, p-PI3K, p-AKT, p-S6K1, and p-eIF4B. GAPDH was used to normalize the data. 740Y-P (30 μM) is an activator of the PI3K/Akt signaling pathway. All data are expressed as mean ± SEM, unpaired Student’s t‐test, Control+Gas vs Control , Glucose+740Y-P vs Glucose. NS: Not significant.


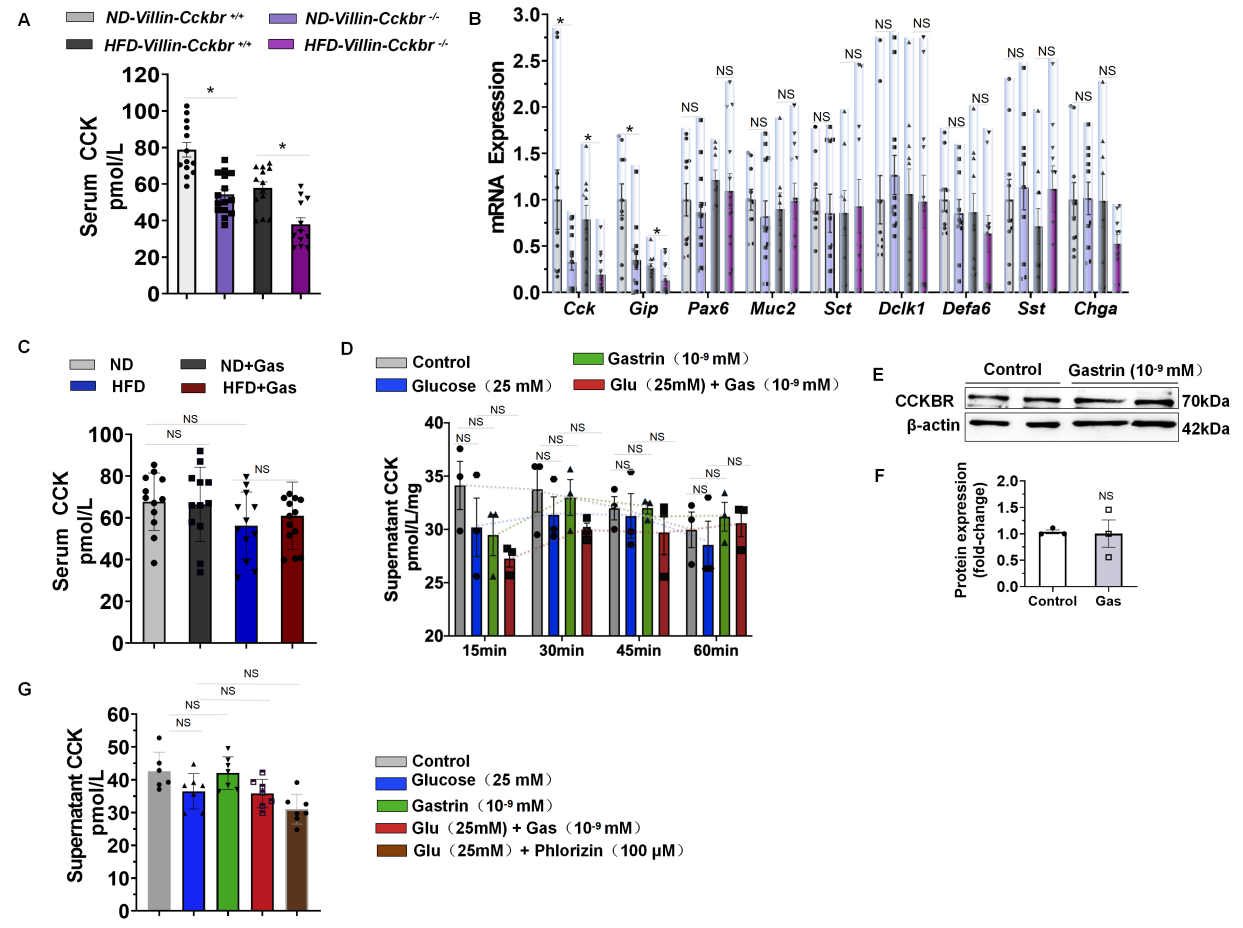


**Figure S7: Gastrin-SiO_2_ microspheres indirectly increase the secretion of incretins**. **A**: Serum levels of CCK (cholecystokinin) in *ND-Villin-Cckbr^+/+^* mice, *ND-Villin-Cckbr^-/-^* mice, *HFD-Villin-Cckbr^+/+^* mice, and *HFD-Villin-Cckbr^-/-^* mice, n=12-14/group; **B**: Cell-specific regulator genes: *Cck* (I cells), *Gip* (K cells), *Pax6* (EEC cells), *Muc2* (Goblet cells), *Sct* (S cells), *Dclk1* (Tuff cells), *Defa6* (Paneth cells), *Sst* (δ cells), and *Chga* (enterochromaffin cells). **C**: Serum levels of CCK in C57BL/6J mice from the ND group, ND+Gas group, HFD group, and HFD+Gas group, n = 12/group; **D**: CCK levels in supernatant were measured by ELISA, and final concentration divided by isolated intestine overall protein concentration (pmol/L/prot), All data are expressed as mean ± SEM, Two way ANOVA, post hoc Scheffe test (A-D); **E-F**: Western blots and quantification of gastrin receptor (CCKBR) in a human enteroendocrine cell line (NCI-H716); **G**: CCK level in cell culture medium (supernatant). All data are expressed as mean ± SEM, significant differences tested by one-way ANOVA, post hoc Scheffe test(G), *P<0.05; NS = not significant.


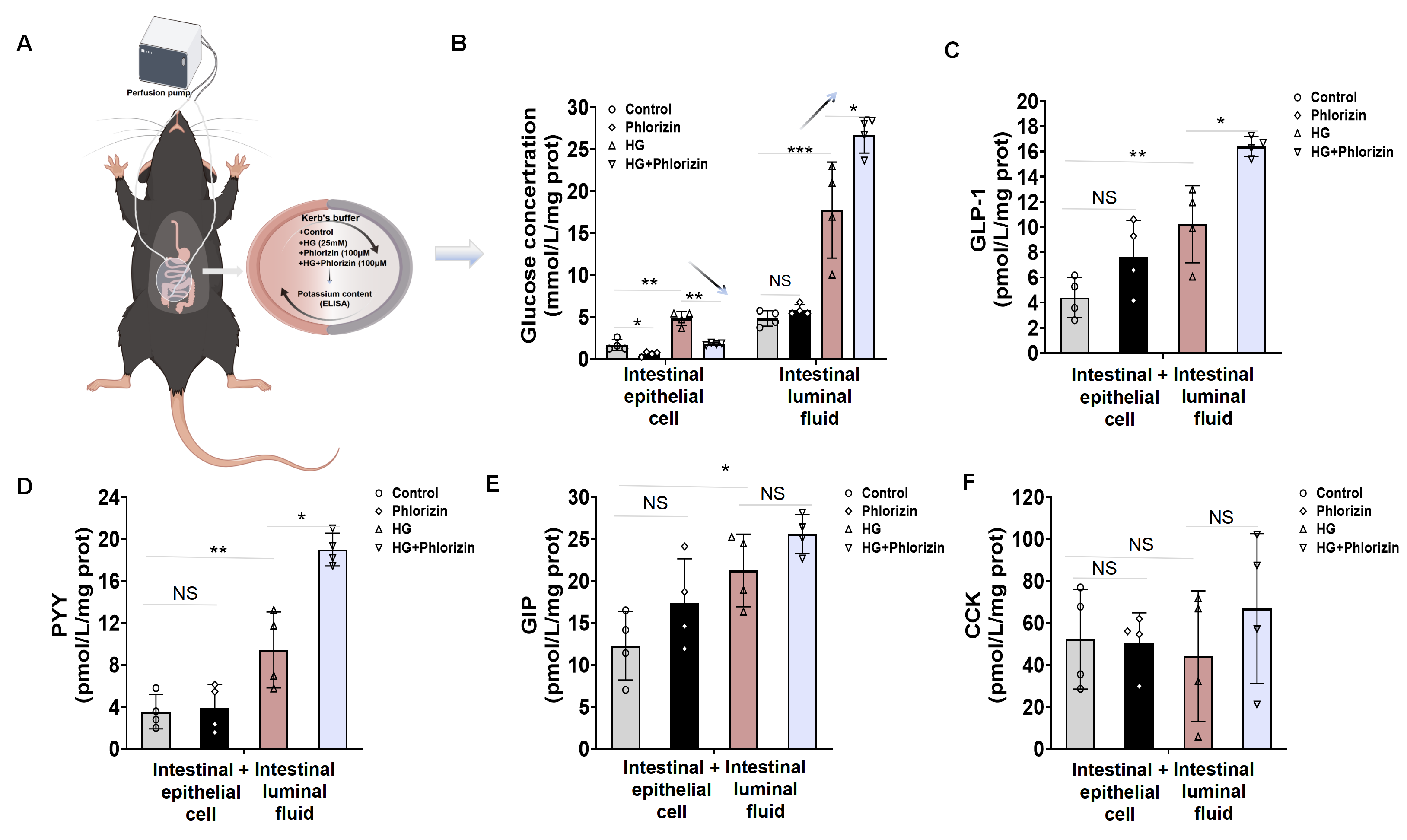


**Figure S8: SGLT1 inhibitor rescue intestinal CCKBR knockout decreased incretins secretion. A:** Flow diagram of the in-situ intestine perfusion *ex-vivo* experiment. The in-situ intestine were perfused in different media(Control , basal Kerb’s buffer; Phlorizin, Control+100µM phlorizin; HG, High glucose 25 mM; HG+Phlorizin, High glucose+100µM phlorizin) for 30 mins and glucose concentrations and incretins were quantified. **B**:The effects of SGLT1 inhibitor (phlorizin,100µM) on glucose concentration with or without high glucose absorption(25 mM) in intestinal epithelial cell and intestinal luminal in *Villin-Cckbr^-/-^* mice(n=4). Levels of GLP-1 (glucagon-like peptide 1)(**C**), PYY (peptide YY)(**D**), GIP (glucose‐dependent insulinotropic polypeptide)(**E**) and CCK (Cholecystokinin)(**F**) in intestinal epithelial cell and intestinal luminal in *Villin-Cckbr^-/-^* mice(n=4) in-situ intestine perfusion. Two-way ANOVA, post hoc Scheffe test (B-F), HG group vs Control group, HG+ phlorizin group vs HG group, phlorizin group vs Control group, *P<0.05, **P<0.01, ***P<0.001, NS: not significant.
